# Supplementary material for: Regulation of ABCA1 by AMD-Associated Genetic Variants and Hypoxia in iPSC-RPE
Source: Int J Mol Sci. 2022 Mar 16;23(6):3194. doi: 10.3390/ijms23063194 (PMC8953808; doi:10.3390/ijms23063194)
Supplement: Supplementary file 1 [file ijms-23-03194-s001.zip › ijms-1630896-supplementary.pdf]

Supplementary Material:

Regulation of ABCA1 by AMD-associated genetic variants and hypoxia in iPSC-RPE

Florian Peters[1], Lynn J.A. Ebner[1], David Atac[2], Jordi Maggi[2], Wolfgang Berger[2], Anneke I. den Hollander[3,4], Christian Grimm[1,*]

Supplementary Figures

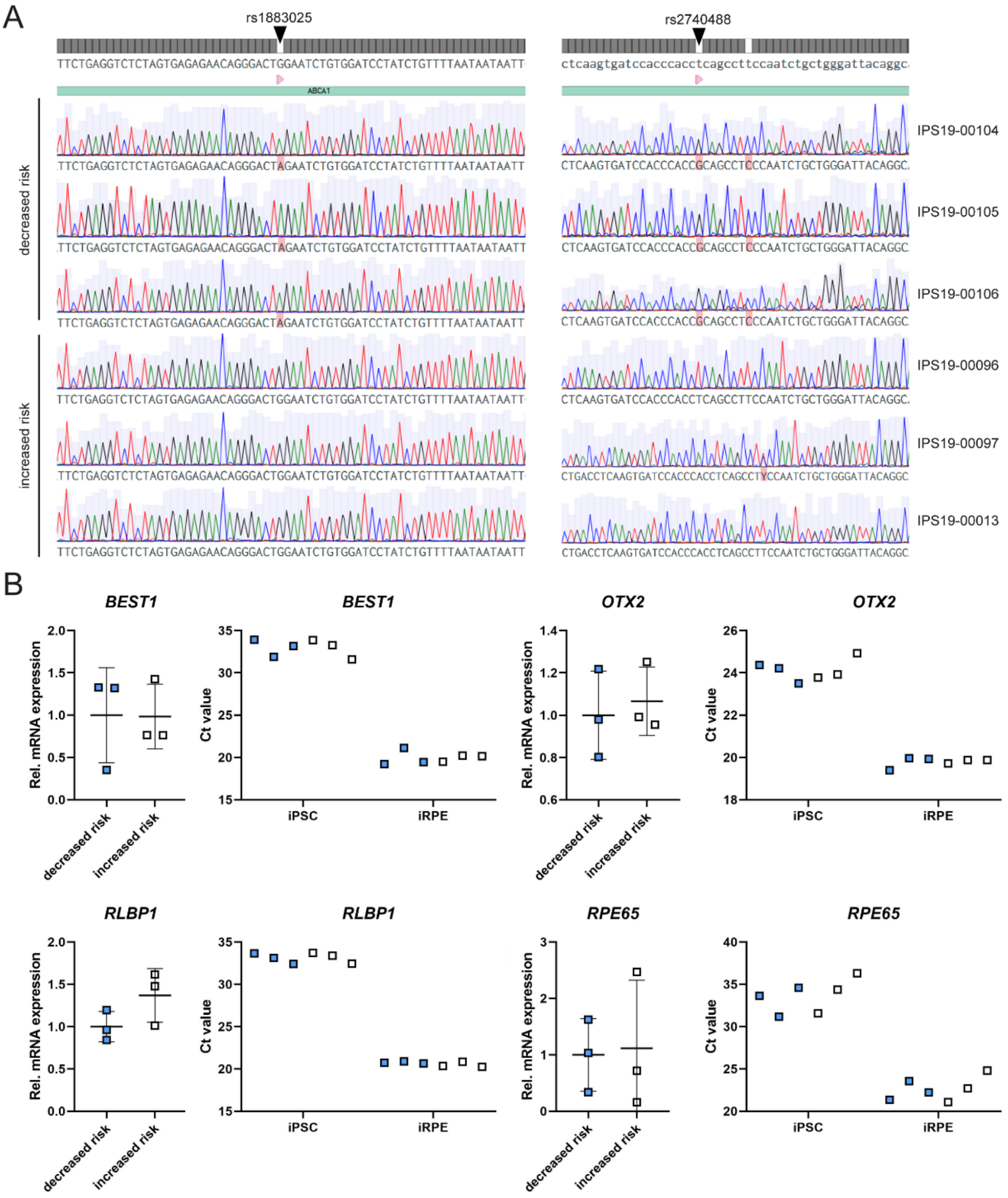

Figure S1
(A) Sequence alignment of the *ABCA1* AMD-associated SNP regions in patient-derived iRPE cells after Sanger sequencing. (B) Relative expression of RPE marker genes *BEST1*, *OTX2*, *RLBP1*, and *RPE65*, and the respective Ct values in patient-derived iRPE. Values were normalized to *RPL28* and decreased risk group. Data are presented as mean ± SD (n = 3). Unpaired Student's t-test.

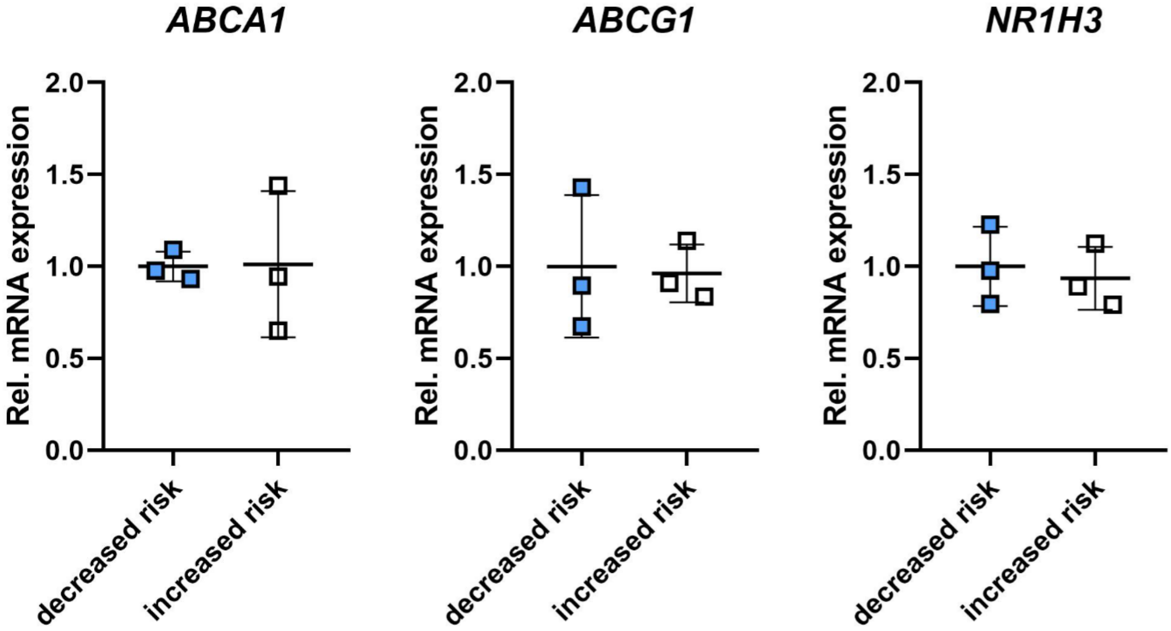

Figure S2
Relative expression of *ABCA1*, *ABCG1* and *NR1H3* in the parental iPSC lines. Shown are means ± SD (n = 3). Unpaired Student’s t-test.

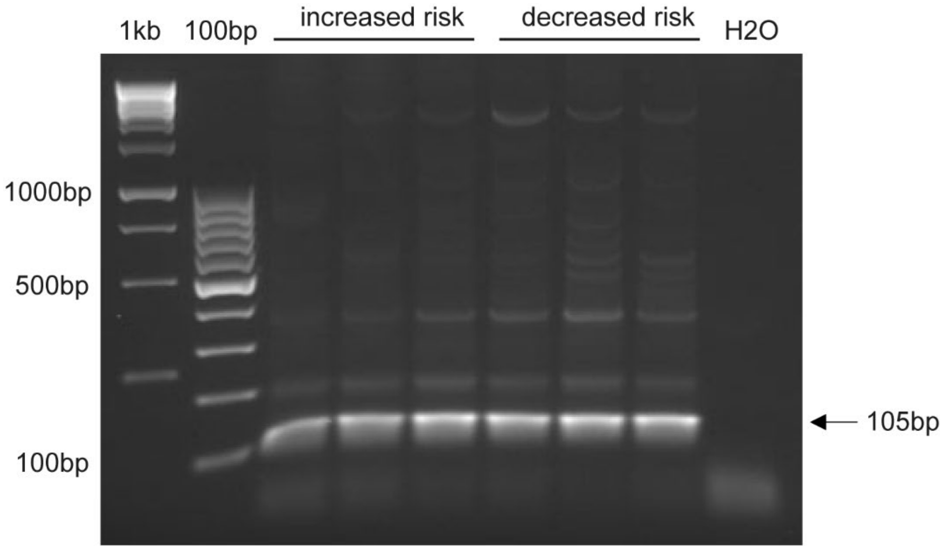

Figure S3
Agarose gel electrophoresis of PCR products from exon 2 to exon 3 of ABCA1 using cDNA from patient-derived iRPE.

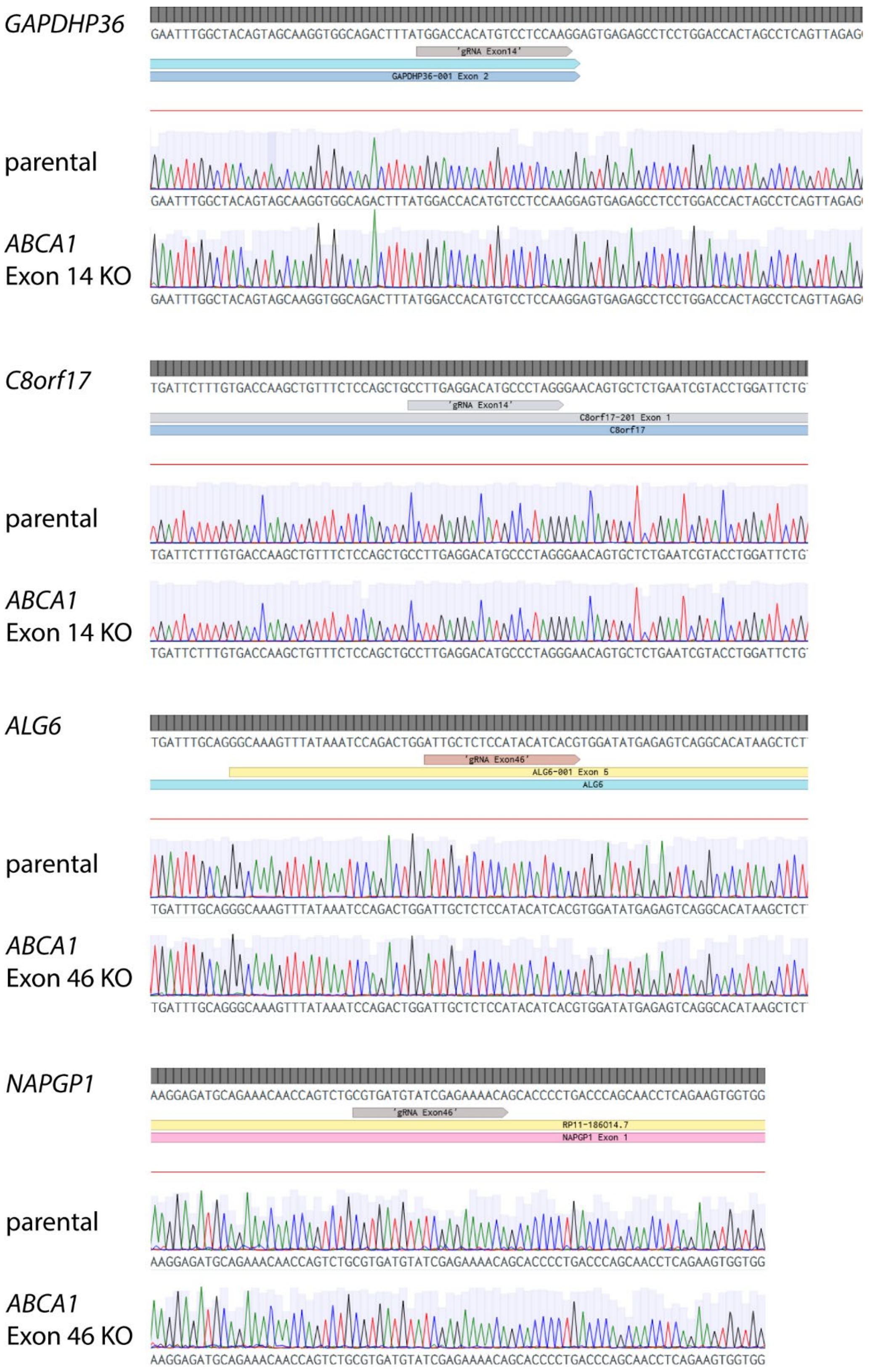

Figure S4
PCR products of potential gRNA off-target regions in parental and ABCA1 KO cell lines were sequenced and aligned. No editing was found in these regions.

Supplementary Tables

Table S1: Off-target prediction for gRNA targeting *ABCA1* exon 14.

Sequence	PAM	Score	Gene	Chromosome	Strand	Position	Mismatches
**CGTACCGCATGTCCTCAAAG**	GGG	100	ENSG00000165029 (*ABCA1*)	chr9	1	107593350	0
**GGTATCAGATGTCCTCAAAG**	GGG	1.01807008		chr7	-1	132243563	4
**TGTTACACATGTCCTCAAAG**	CAG	0.98509615		chr1	1	82586772	4
**AGGTCCTCATGTCCTCAAAG**	AGG	0.97130481		chr1	1	109263797	4
**GGGGCAGCATGTCCTCAAAG**	TGG	0.83997554		chr16	1	11262979	4
**TGTATCACATATCCTCAAAG**	AAG	0.61104935		chr12	-1	32081366	4
**CTCACCTCATCTCCTCAAAG**	AAG	0.59709112		chr1	-1	115603640	4
**CTGACCTCATCTCCTCAAAG**	AAG	0.59709112		chr3	1	135973714	4
**CCTAATGCTTGTCCTCAAAG**	TGG	0.54642985		chr4	1	112593489	4
**TGGACCACATGTCCTCCAAG**	GAG	0.51031067	ENSG00000213158 (*GAPDHP36*)	chr3	1	179930893	4
**CATAGAGCATGCCCTCAAAG**	CAG	0.47552186		chr3	1	53070145	4
**TGTACAGCAAGACCTCAAAG**	GAG	0.46286306		chr3	-1	39547586	4
**CTTACTGGATGTGCTCAAAG**	TGG	0.38795625		chr22	1	21171149	4
**CATATGGCATGTTCTCAAAG**	TAG	0.38438062		chr3	-1	176867798	4
**CCTAGGGCATGTCCTCAAGG**	CAG	0.37509582	ENSG00000250733 (C*8orf17*)	chr8	-1	140945400	4
**CGTCCAGCCTGTCCTCAAAT**	GGG	0.30007957		chr17	-1	72453476	4

Table S2: Off-target prediction for gRNA targeting ABCA1 exon 46.

Sequence	PAM	Score	Gene	Chromosome	Strand	Position	Mismatches
**ATTTTTCTCCATACTTCACG**	AGG	100	ENSG00000165029 (*ABCA1*)	chr9	1	107550301	0
**CTTTCTCTCCTTACTTCACG**	GAG	1.71463415		chr8	1	342405	3
**AGTATTCTCCATCCTTCACG**	GGG	1.24416244		chr1	1	45056082	3
**CTTTTTCTCCCTACTTCACC**	AAG	1.10207143		chr3	1	130088387	3
**TTTTTGCTTCATACTTCACG**	GAG	1.05934314		chr13	1	67413592	3
**TTTCTTCCCCATACTTCACC**	TAG	0.91138804		chr12	1	57816291	4
**TGTTCTCTCCATACTTCACT**	TGG	0.90034091		chr2	1	118791463	4
**CCTTTTCTCTATACTTCACA**	CAG	0.88271165		chr12	1	108079423	4
**ATTGCTCTCCATACATCACG**	TGG	0.86159052	ENSG00000088035 (*ALG6*)	chr1	1	63870166	3
**ATATATCTCAATACTTCACT**	TGG	0.77979666		chr6	1	48925107	4
**ACTGTTCTCAAAACTTCACG**	CAG	0.75787923		chr11	1	132214812	4
**ATTTTTGGCCATACTTCAAG**	GGG	0.72094444		chr11	-1	127480827	3
**ATCTGTCGCCATACTTCAAG**	TGG	0.60463217		chr6	-1	46690467	4
**ATTCATCTCTATACTTCAAG**	TGG	0.5586674		chr1	-1	69667850	4
**CTTTCTATCCAGACTTCACG**	AAG	0.55680384		chr4	1	35118646	4
**GTTTTTCTCCATTCTTCAGG**	AGG	0.54756383		chr15	-1	69231745	3
**TTTTTCCCCCATACTTCACA**	GAG	0.53818282		chrX	-1	117467965	4
**GTTTCTCTCCTTACTTCACT**	CAG	0.5185454		chr3	-1	109888182	4
**ATTGCTCTACATACTTCACA**	AAG	0.50147994		chr1	-1	146310934	4
**ATTGCTCTACATACTTCACA**	AAG	0.50147994		chr16	1	70873755	4
**ACTGTTTTCCATACTTGACG**	AAG	0.49564931		chr12	1	19911351	4
**TGTTTTCTCGATACATCACG**	CAG	0.47531068	ENSG00000271408 (*NAPGP1*)	chr10	-1	90459619	4
**GTCTTTCTCCAGACTTCACA**	GAG	0.47102235		chr10	-1	69335630	4

Supplementary Data Sheets

Pluripotency characterization of generated iPSC lines

Stem Cell Technology Center Genetica

Certificate of Analysis 2020

Invoice number: SCTC2019-00088

Name investigator: Christian Grimm
Cell line number: IPS19-00104
Project name: Zurich

Table 1: Information on the reprogrammed cell line

**Information cell line:**	
Product description	EBVs nucleofected with episomal vectors containing the genes OCT3/4, SOX2, KLF4, L-MYC, LIN28
Parental cell line	037362
Parental cell type	EBV immortalized B-lymphocytes
Diagnosis	AMD-O
Mutation	N/A*
Number of clones	3
Passage (P) of iPSCs reported at submission	P10
Culture medium	Essential 8 Flex medium
Culture coating	Matrigel
Feeders during reprogramming	Mouse Embryonic Fibroblasts (MEFs)
Passage method	0.5 mM EDTA
Protocols in Q-portal	046588; 046591

Table 2: Information on the characterization of the reprogrammed cell line

**Test description:**	**Test method:**	**Test specification:**	**Result:**
Activation of stem cell markers	qPCR	Upregulation of *SOX2, LIN28, NANOG, DNMT3B* compared with EBVs	Pass
Expression of stem cell markers	Immunocytochemistry	Expression of OCT4, NANOG, SSEA4, TRA-1-81	Pass
Mycoplasma	PCR	Negative	Pass
Three lineage differentiation	Differentiation assay	Upregulation of germlayer-specific genes	Pass
hPSC genetic analysis	qPCR	Detection of recurrent chromosomal abnormalities	See results in last page

*N/A: Not applicable

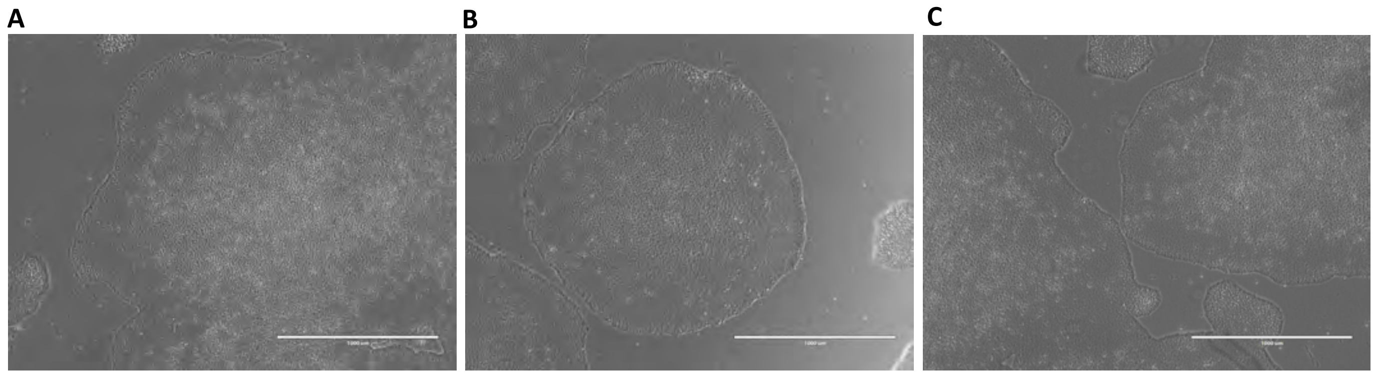

Figure 1: Cells prior to freezing. A - C, clone 1, clone 2 and clone 3, respectively at P10. Scale bar = 1000 µm.

Activation of stem cell markers

All clones were assessed for activation of stem cell markers before freezing. RNA was isolated and gene expression was assessed by quantitative reverse transcription PCR. Ct values were normalized with the housekeeping gene GUSB (set at 1).

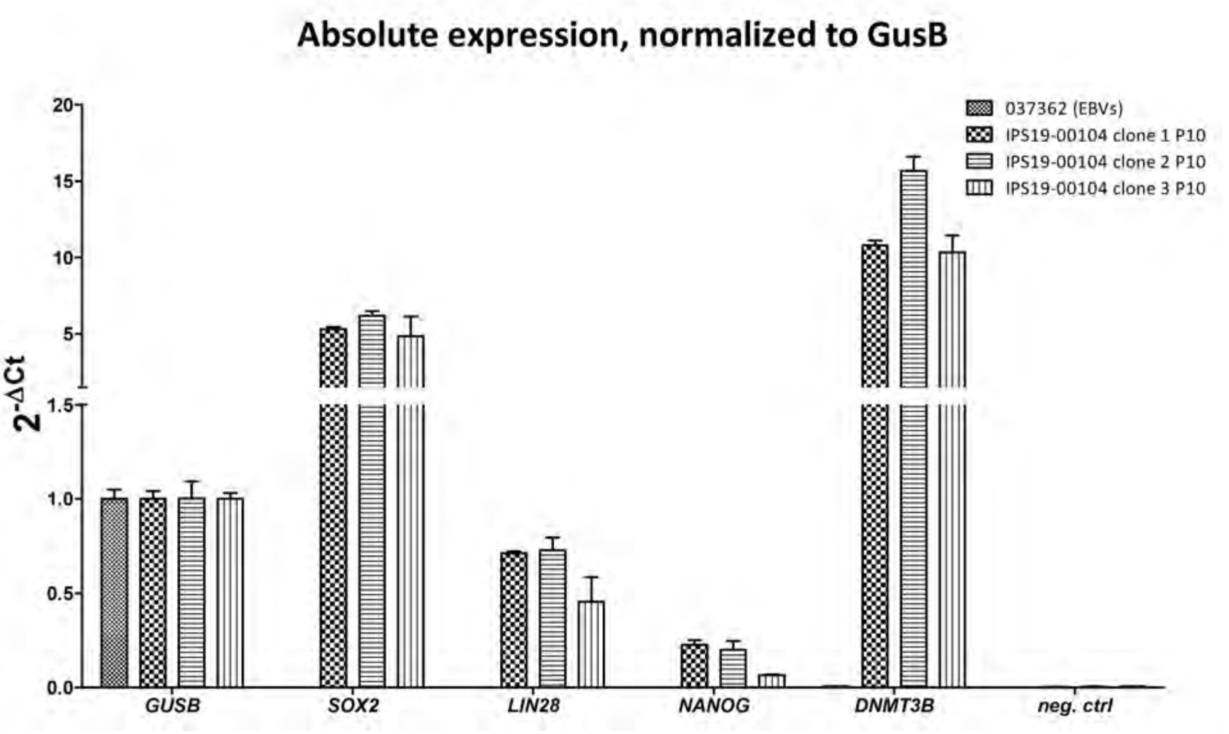

Figure 2: Gene expression of three iPSC clones compared with the parental EBVs (ΔCt).

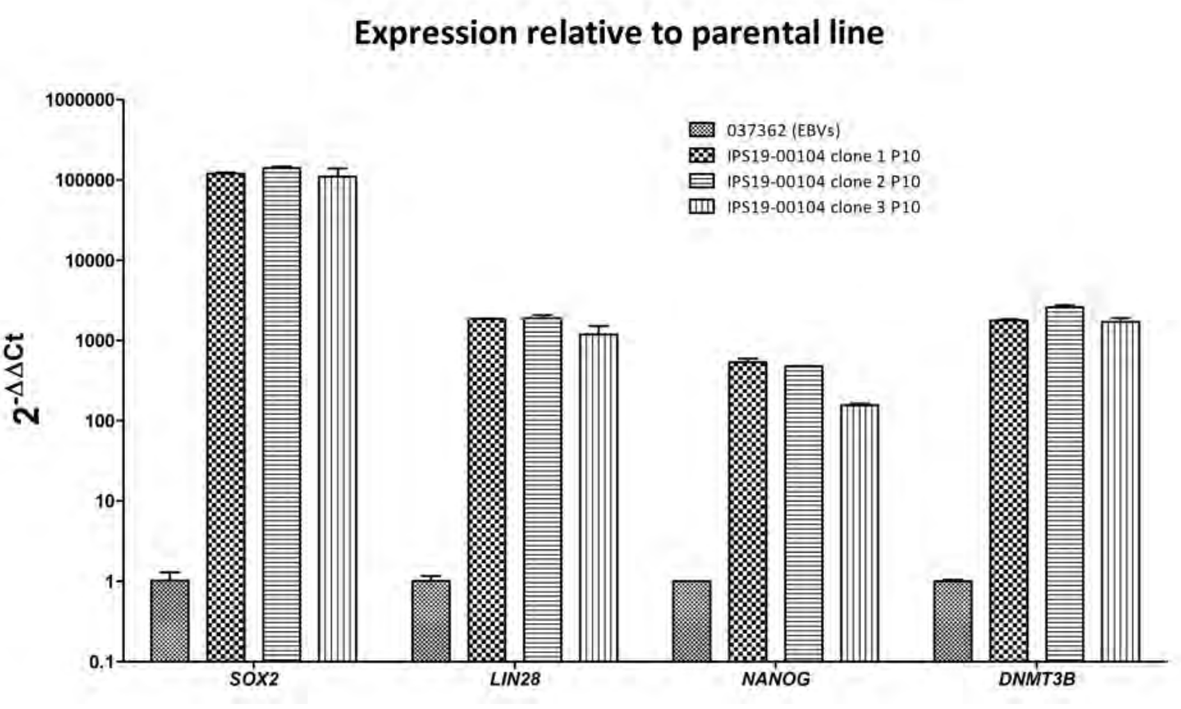

Figure 3: Pluripotency gene upregulation after reprogramming (ΔΔCt). The expression fold difference of the iPSCs is relative to the parental EBVs.

Expression of stem cell markers

Undifferentiated iPSC clones were stained for the nuclear markers NANOG and OCT4 and surface antigens SSEA4 and TRA-1-81. All markers are expressed in human pluripotent stem cells.

A. IPS19-00104 clone 1

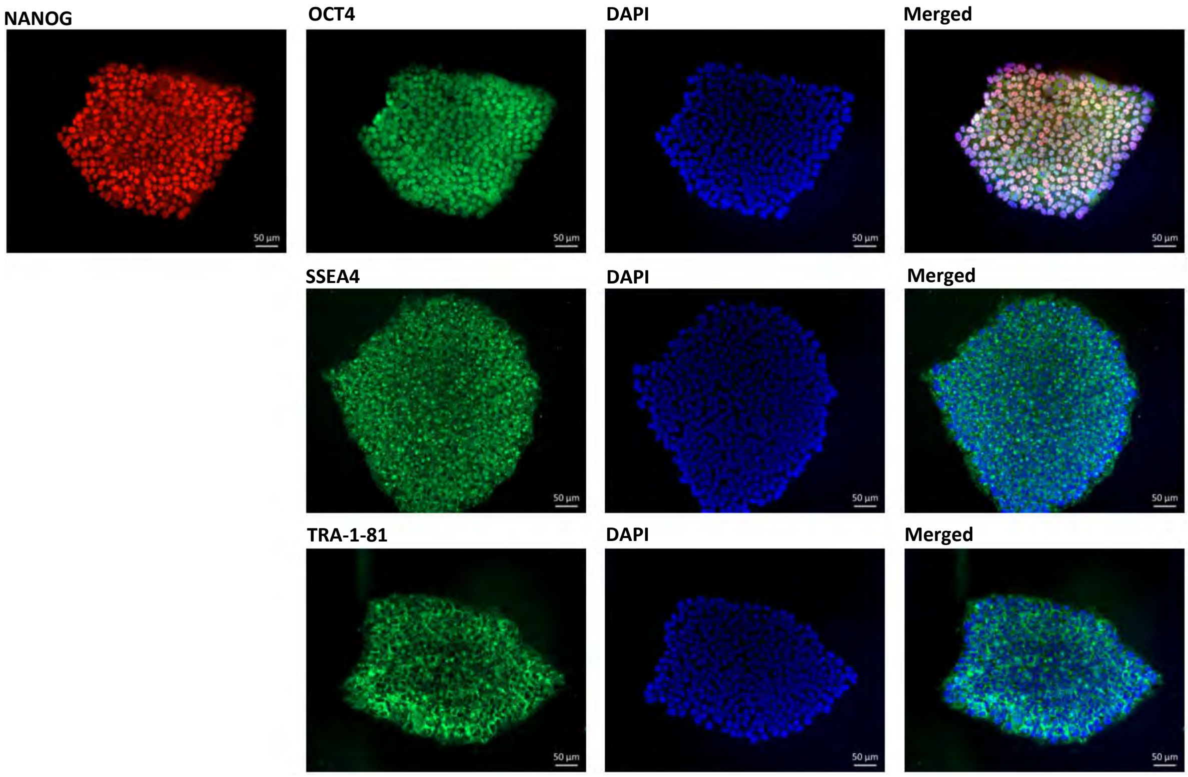

B. IPS19-00104 clone 2

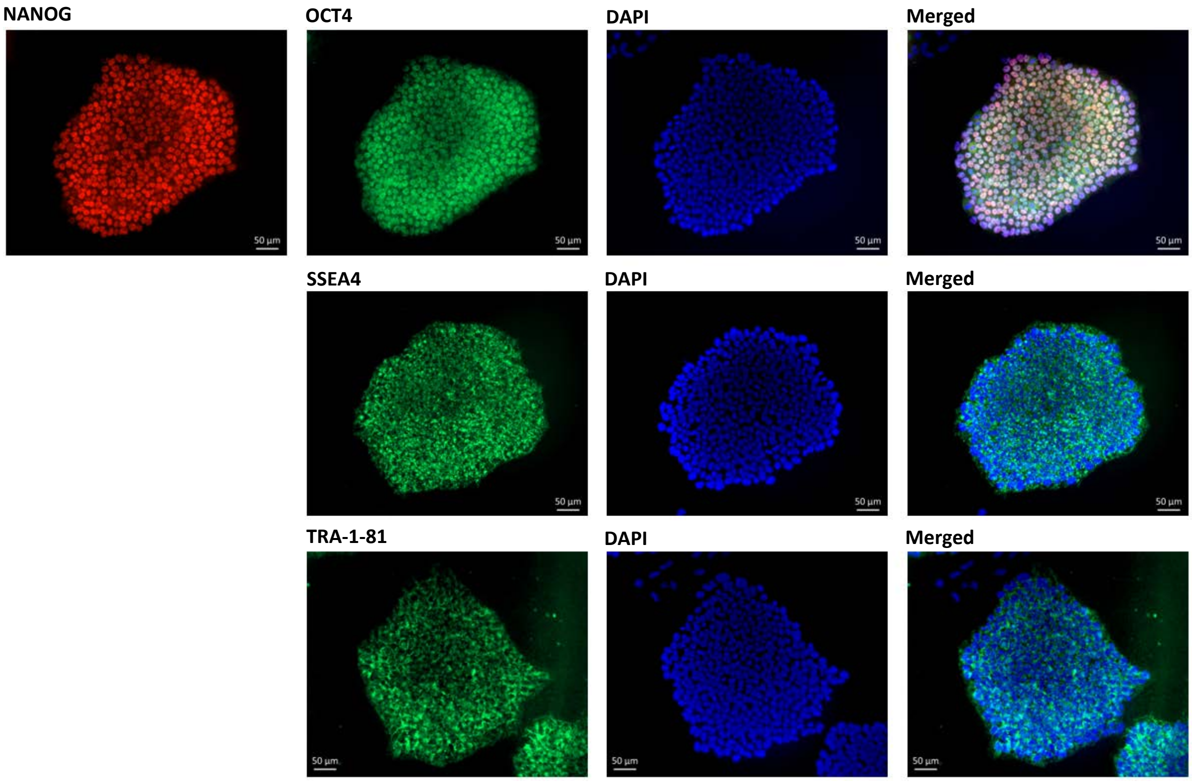

C. IPS19-00104 clone 3

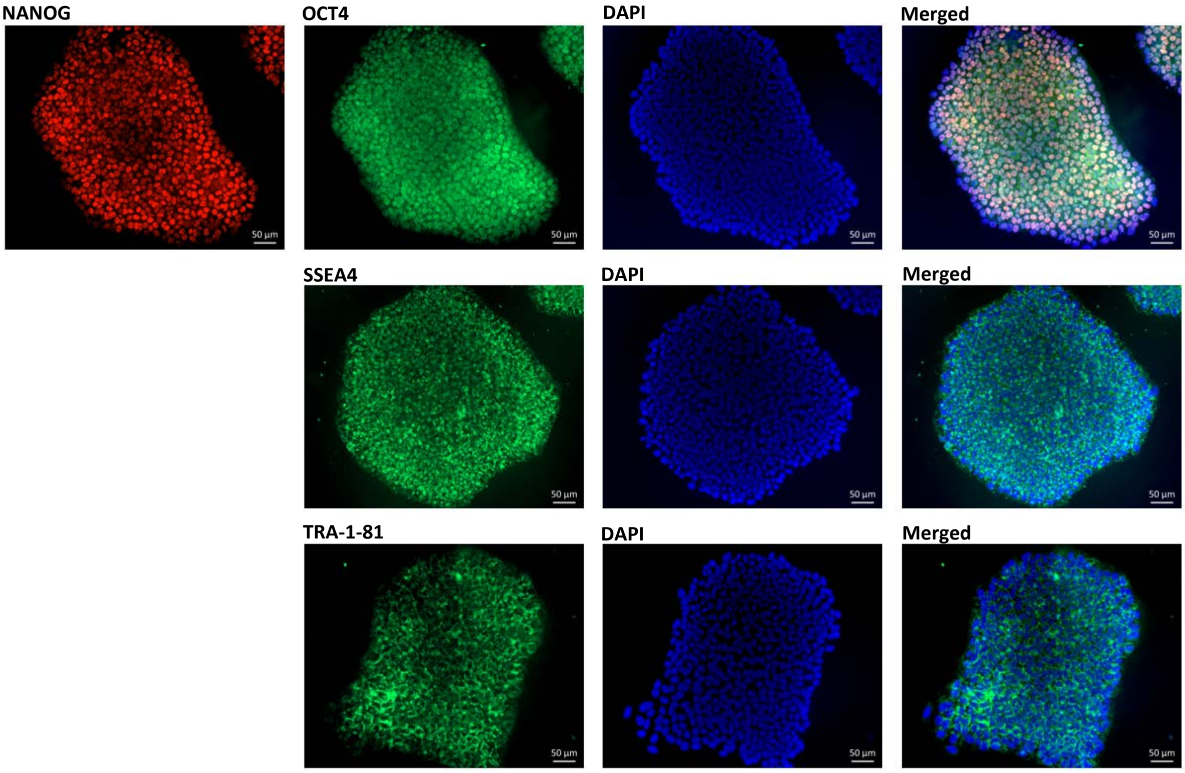

Figure 4: Immunofluorescence staining of the iPSC clones with pluripotency markers.

Three germ layer differentiation

IPS19-00104 clone 1 was differentiated into the endodermal, mesodermal and ectodermal germ layers. RNA was isolated and gene expression was checked by qPCR. Ct values are normalized with the housekeeping gene GUSB (set at 1). For each lineage two genes were assessed (Table 3). The differentiated cells were also stained for lineage-specific markers (Table 4).

Table 3: qPCR markers for three lineage differentiation

Lineage	Marker
Endoderm	FOXA2, SOX17
Mesoderm	Brachyury, HAND1
Ectoderm	PAX6, NCAM1

Table 4: ICC markers for three lineage differentiation

Lineage	Marker
Endoderm	SOX17
Mesoderm	NCAM1
Ectoderm	NESTIN

Endoderm

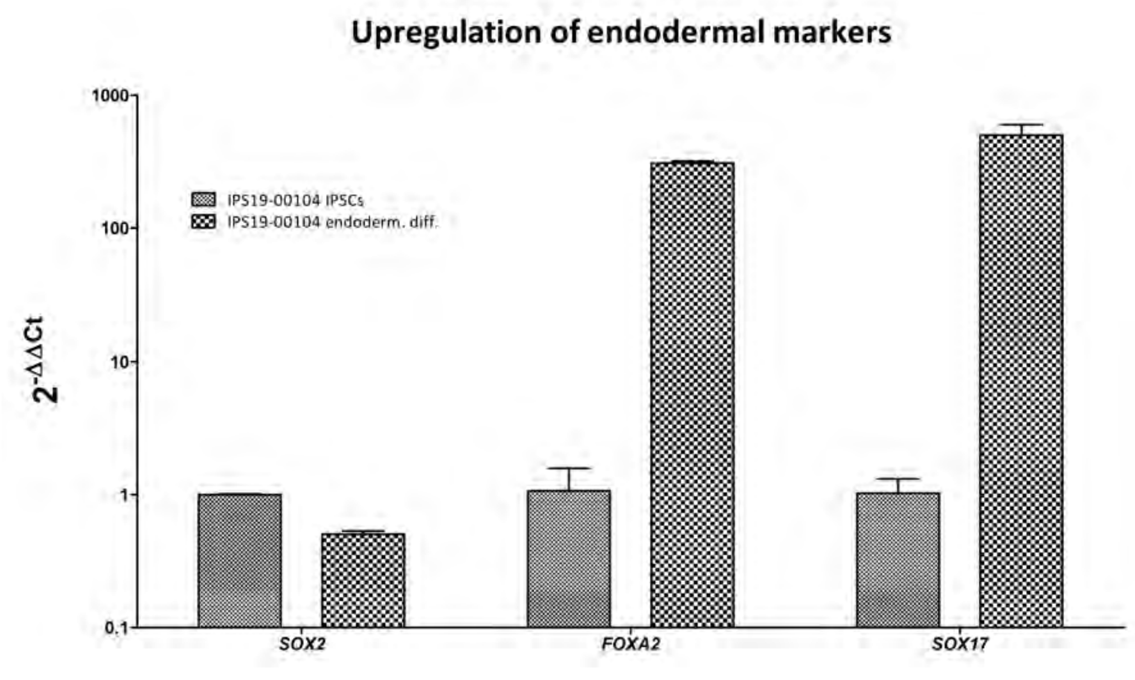

Figure 5: Expression fold difference of endoderm-specific genes in differentiated cells, compared with undifferentiated iPSCs. *SOX2* was used as a reference for pluripotency.

Mesoderm

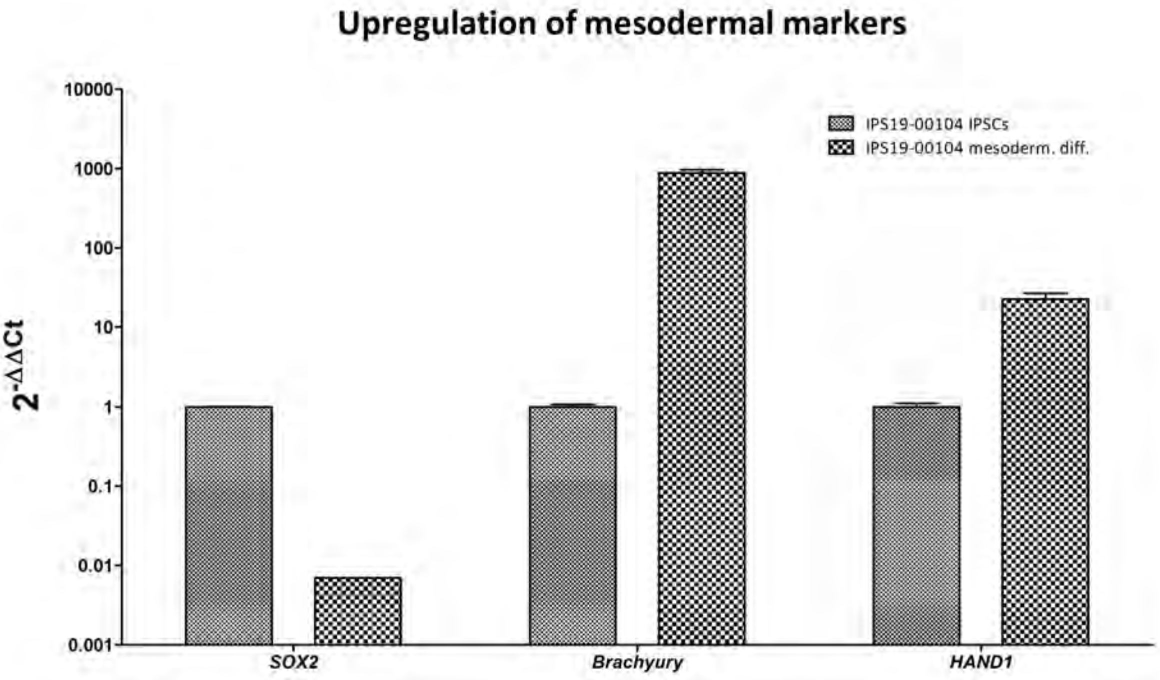

Figure 6: Expression fold difference of mesoderm-specific genes in differentiated cells, compared with undifferentiated iPSCs. *SOX2* was used as a reference for pluripotency.

Ectoderm

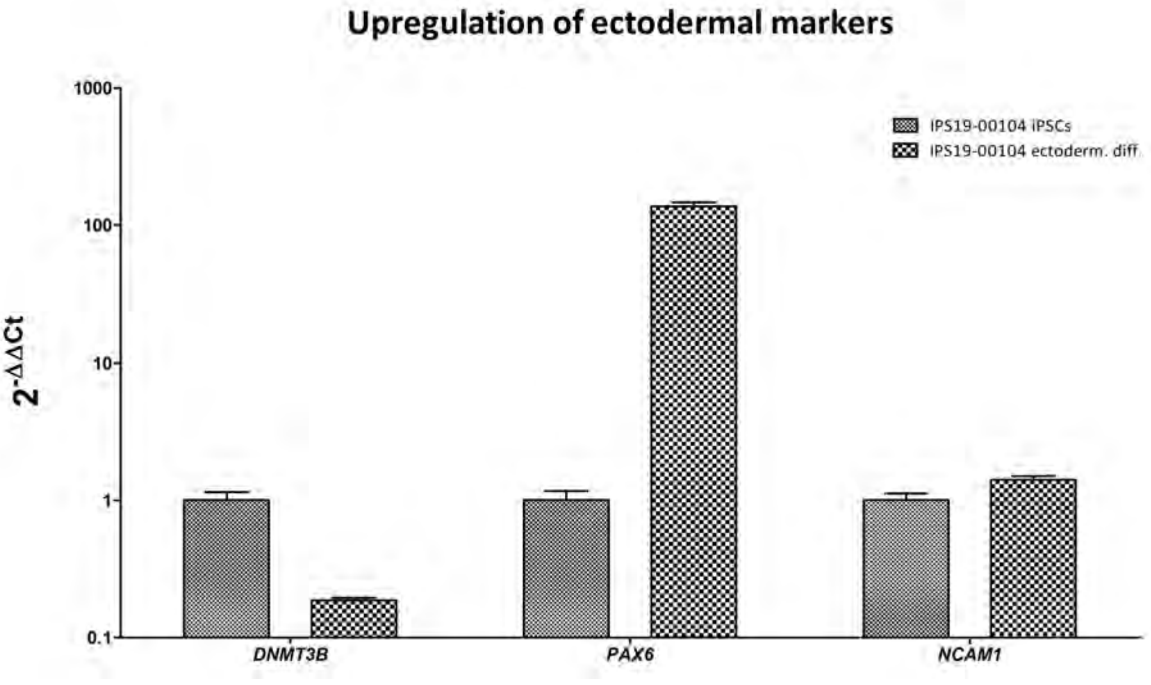

Figure 7: Expression fold difference of ectoderm-specific genes in differentiated cells, compared with undifferentiated iPSCs. *DNMT3B* was used as a reference for pluripotency.

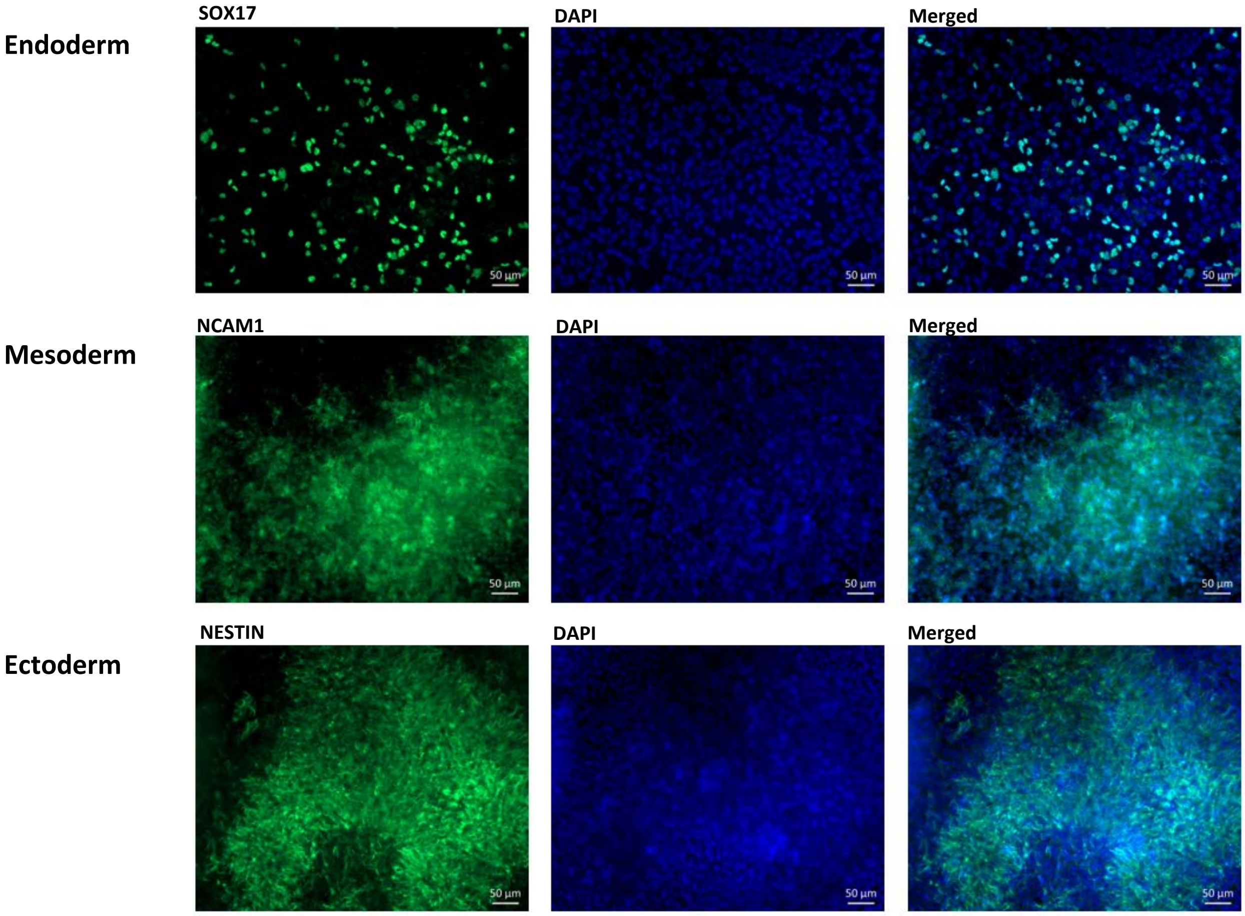

Figure 8: Immunofluorescence staining of differentiated cells showing positive signal of germlayer-specific markers.

Genetic analysis

DNA was isolated from three iPSC clones and the majority of recurrent chromosomal abnormalities reported in human embryonic stem cells and iPSCs was analysed.

Sample	Sex	Status
IPS19-00104 clone 1 p10	Male	Normal (Sex Difference)
IPS19-00104 clone 2 p10	Male	Possibly Abnormal
**IPS19-00104 clone 3 p10**	**Male**	**Abnormal**

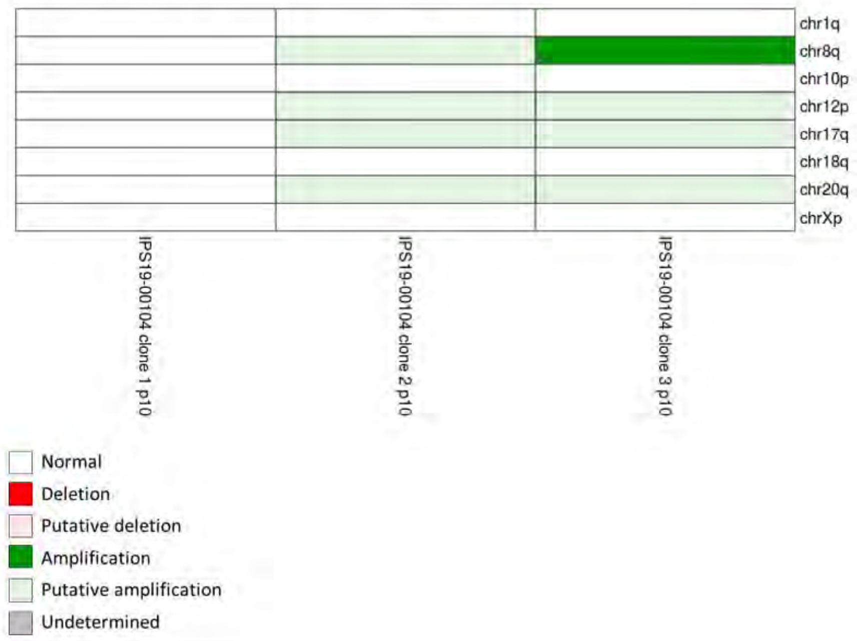

Figure 9: Summary of the genetic analysis

For further experiments it is suggested to use IPS19-00104 clone 1. It is suggested to check IPS19-00104 clone 2 and 3 at a later passage to assess whether there is indeed a mutant clone in the culture that expands over time.

More detailed results are on request.

Stem Cell Technology Center Genetica

Certificate of Analysis 2020

Invoice number: SCTC2019-00090

Name investigator: Christian Grimm
Cell line number: IPS19-00105
Project name: Zurich

Table 1: Information on the reprogrammed cell line

Information cell line:	
Product description	EBVs nucleofected with episomal vectors containing the genes OCT3/4, SOX2, KLF4, L-MYC, LIN28
Parental cell line	044865
Parental cell type	EBV immortalized B-lympocytes
Diagnosis	AMD
Mutation	N/A*
Number of clones	3
Passage (P) of iPSCs reported at submission	P10
Culture medium	Essential 8 Flex medium
Culture coating	Matrigel
Feeders during reprogramming	Mouse Embryonic Fibroblasts (MEFs)
Passage method	0.5 mM EDTA
Protocols in Q-portal	046588; 046591

Table 2: Information on the characterization of the reprogrammed cell line

Test description:	Test method:	Test specification:	Result:
Activation of stem cell markers	qPCR	Upregulation of *SOX2, LIN28, NANOG, DNMT3B* compared with EBVs	Pass
Expression of stem cell markers	Immunocytochemistry	Expression of OCT4, NANOG, SSEA4, TRA-1-81	Pass
Mycoplasma	PCR	Negative	Pass
Three lineage differentiation	Differentiation assay	Upregulation of germlayer-specific genes	Pass
hPSC genetic analysis	qPCR	Detection of recurrent chromosomal abnormalities	See results in last page

*N/A: Not applicable

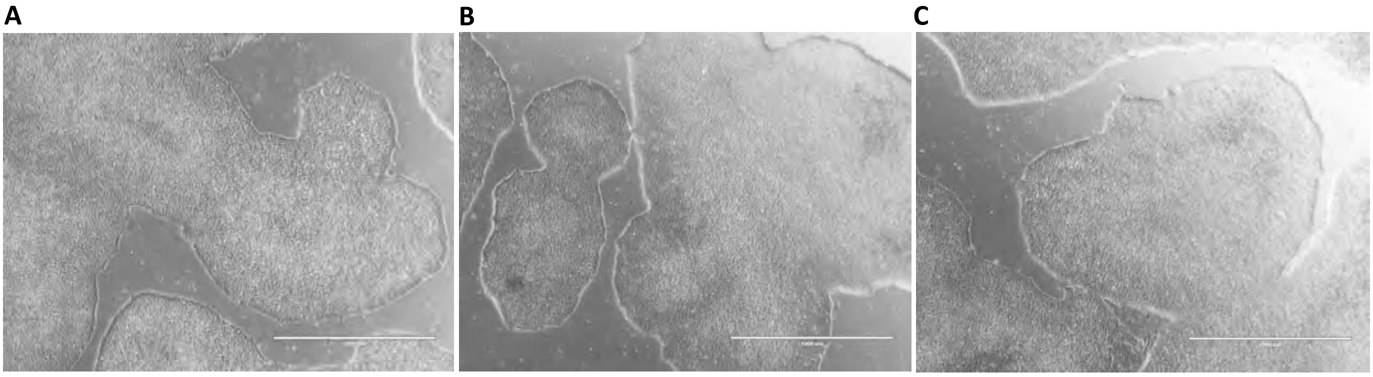

Figure 1: Cells prior to freezing. A - C, clone 1, clone 2 and clone 3, respectively at P10. Scale bar = 1000 µm.

Activation of stem cell markers

All clones were assessed for activation of stem cell markers before freezing. RNA was isolated and gene expression was assessed by quantitative reverse transcription PCR. Ct values were normalized with the housekeeping gene GUSB (set at 1).

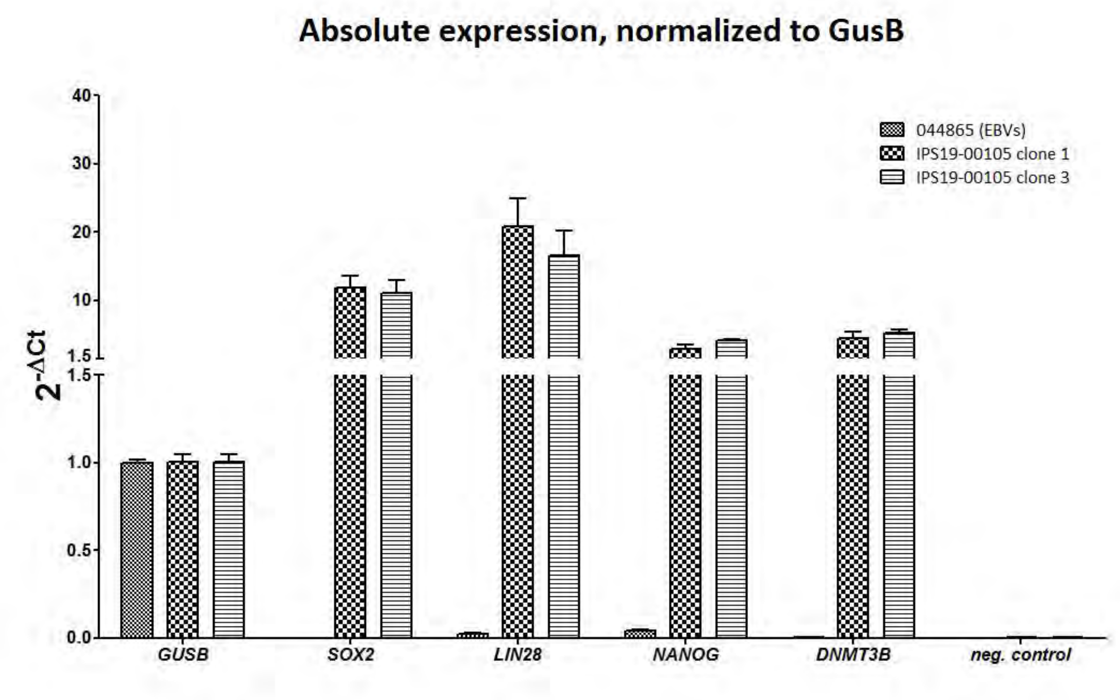

Figure 2: Gene expression of three iPSC clones compared with the parental EBVs (ΔCt).

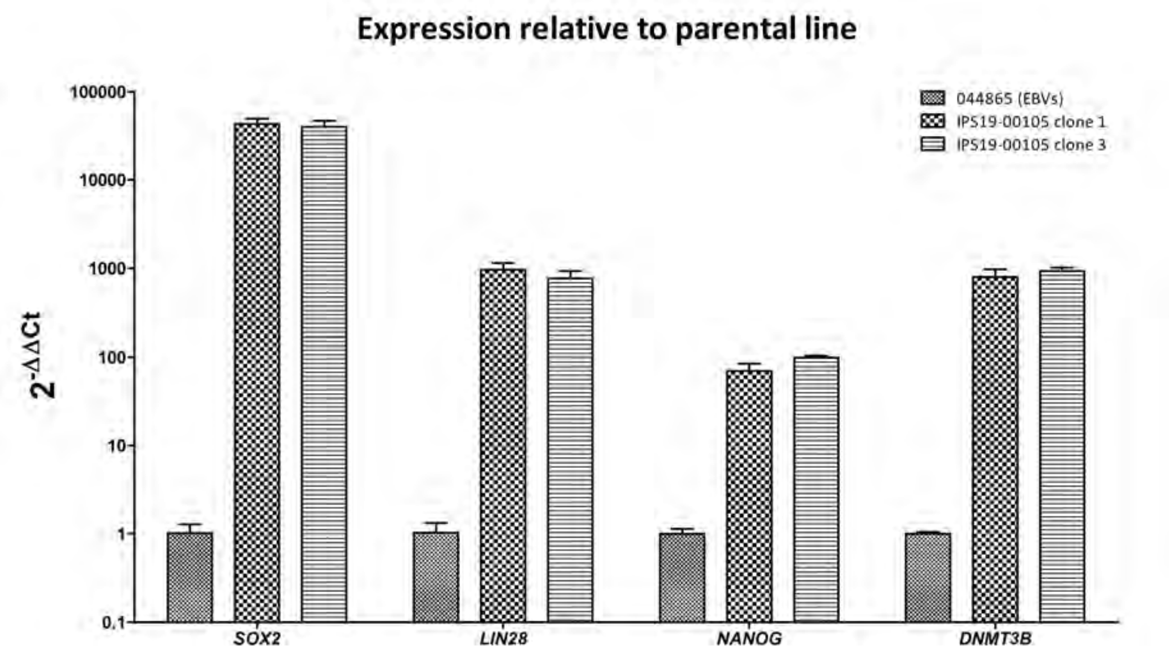

Figure 3: Pluripotency gene upregulation after reprogramming (ΔΔCt). The expression fold difference of the iPSCs is relative to the parental EBVs.

Expression of stem cell markers

Undifferentiated iPSC clones were stained for the nuclear markers NANOG and OCT4 and surface antigens SSEA4 and TRA-1-81. All markers are expressed in human pluripotent stem cells.

A. IPS19-00105 clone 1

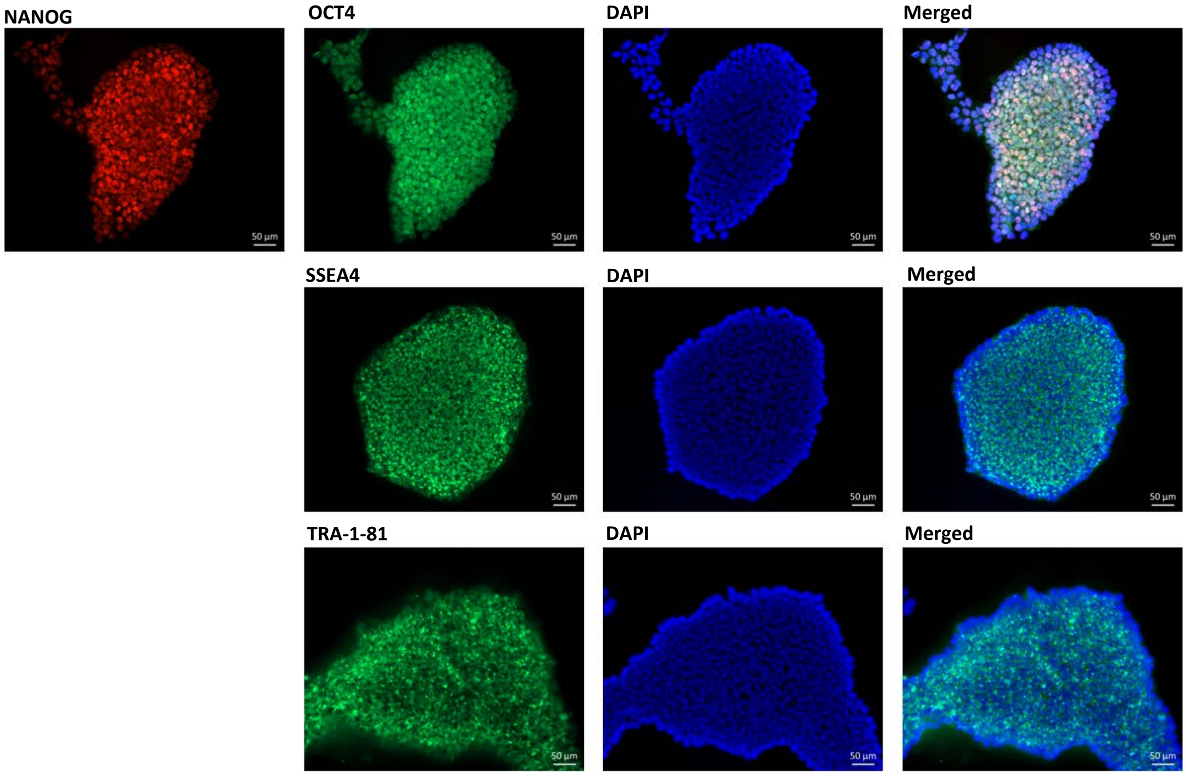

B. IPS19-00105 clone 2

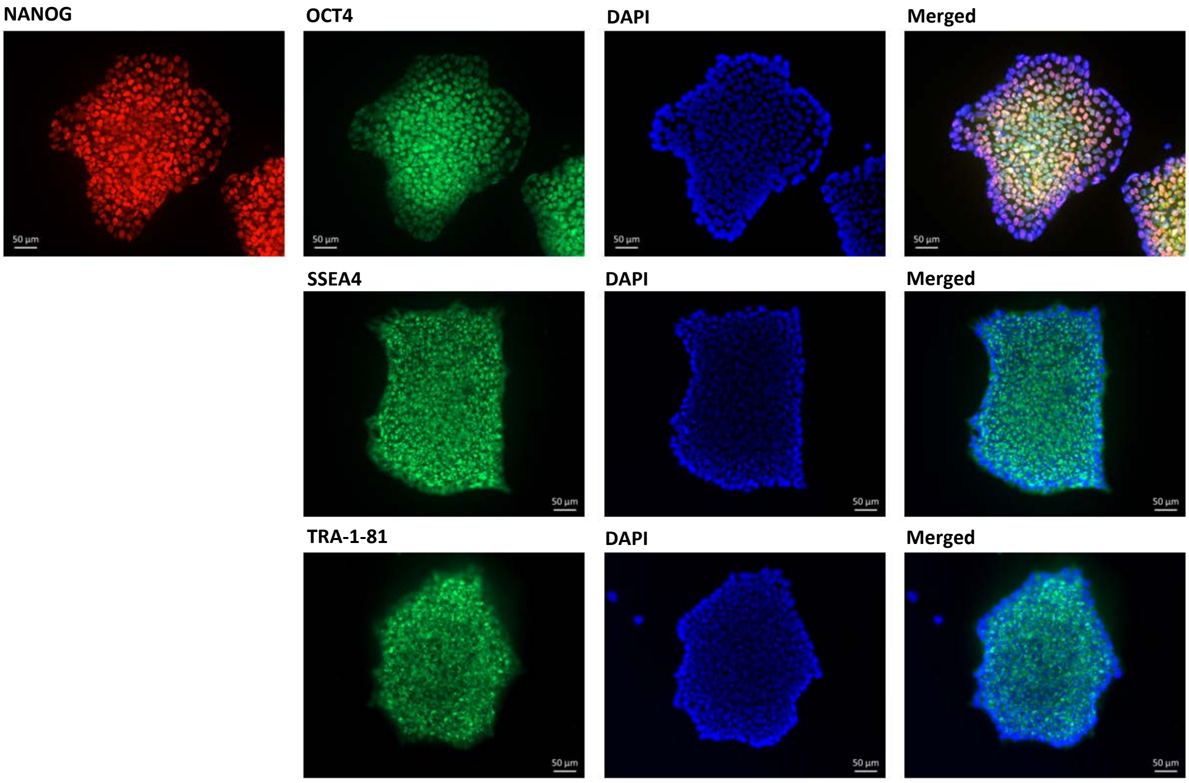

C. IPS19-00105 clone 3

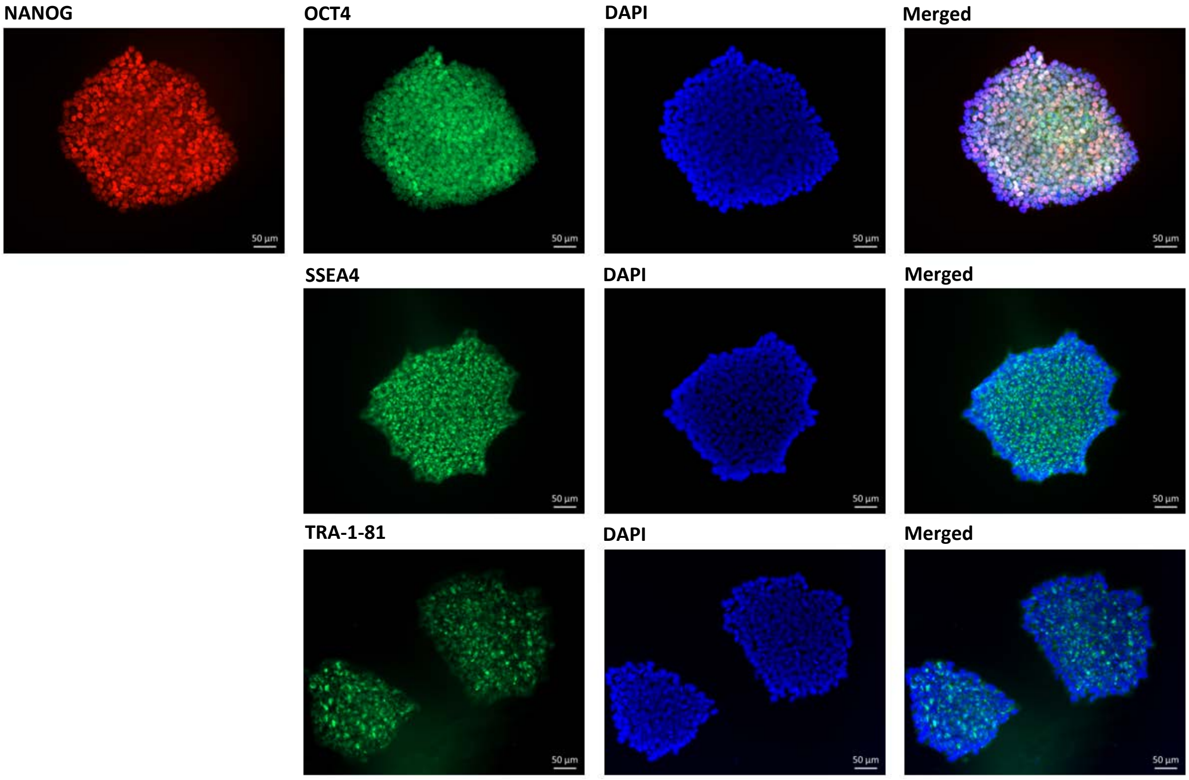

Figure 4: Immunofluorescence staining of the iPSC clones with pluripotency markers.

Three germ layer differentiation

IPS19-00105 clone 1 was differentiated into the endodermal, mesodermal and ectodermal germ layers. RNA was isolated and gene expression was checked by qPCR. Ct values are normalized with the housekeeping gene GUSB (set at 1). For each lineage two genes were assessed (Table 3). The differentiated cells were also stained for lineage-specific markers (Table 4).

Table 3: qPCR markers for three lineage differentiation

Lineage	Marker
Endoderm	FOXA2, SOX17
Mesoderm	Brachyury, HAND1
Ectoderm	PAX6, NES

Table 4: ICC markers for three lineage differentiation

Lineage	Marker
Endoderm	SOX17
Mesoderm	NCAM1
Ectoderm	NESTIN

Endoderm

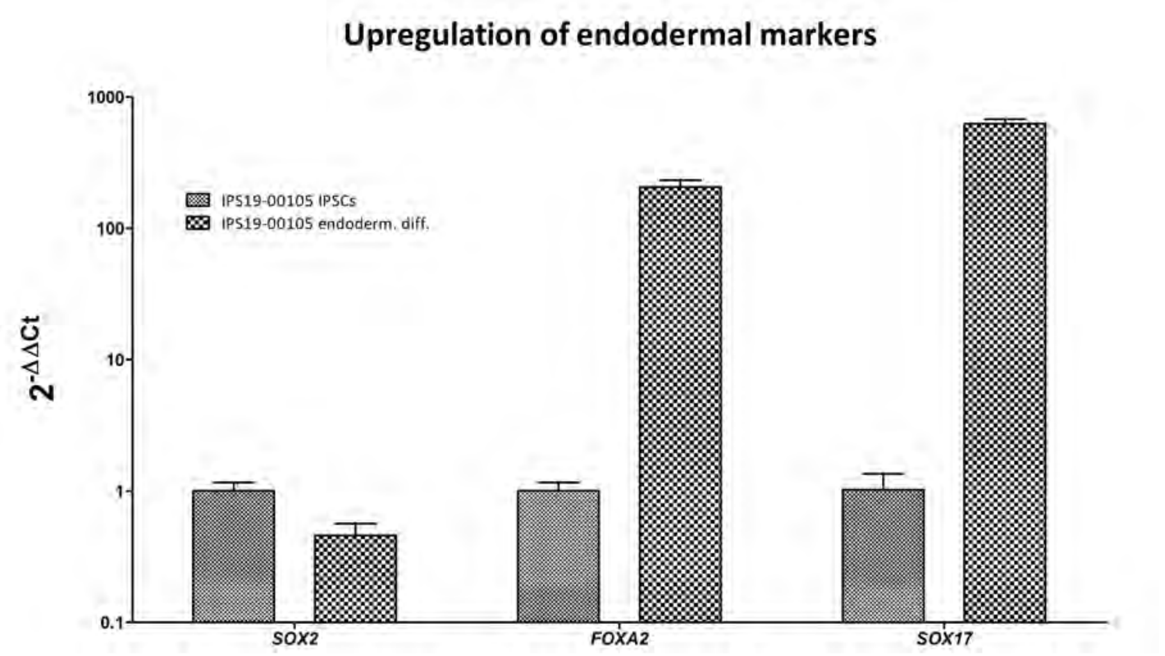

Figure 5: Expression fold difference of endoderm-specific genes in differentiated cells, compared with undifferentiated iPSCs. *SOX2* was used as a reference for pluripotency.

Mesoderm

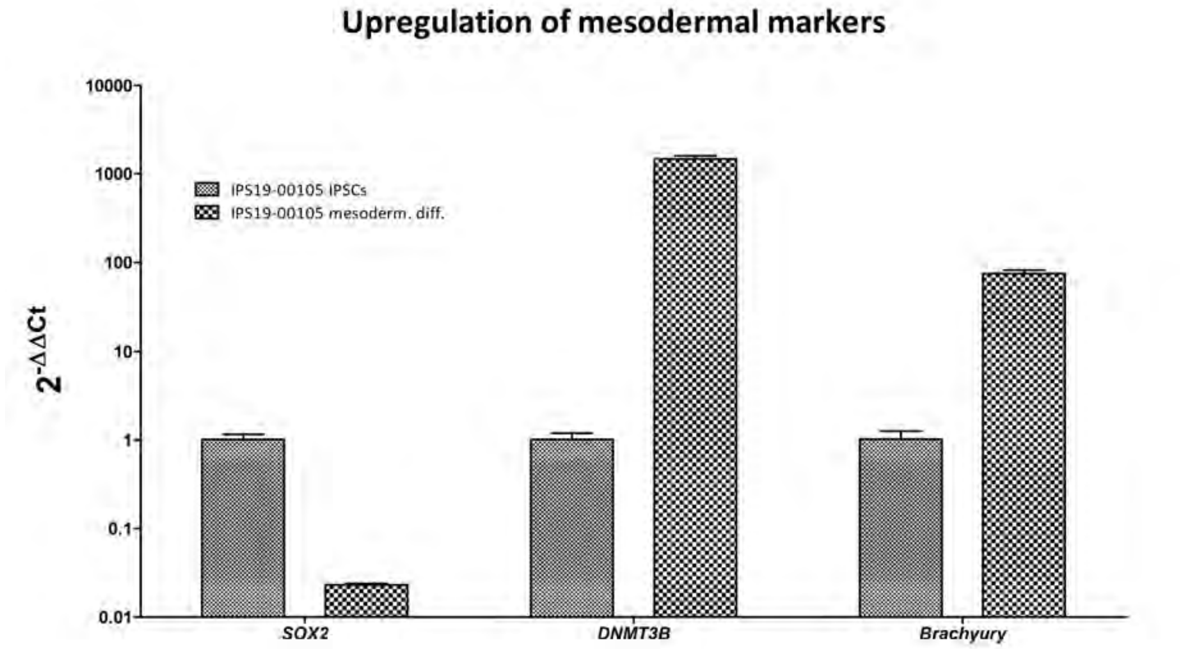

Figure 6: Expression fold difference of mesoderm-specific genes in differentiated cells, compared with undifferentiated iPSCs. *SOX2* was used as a reference for pluripotency.

Ectoderm

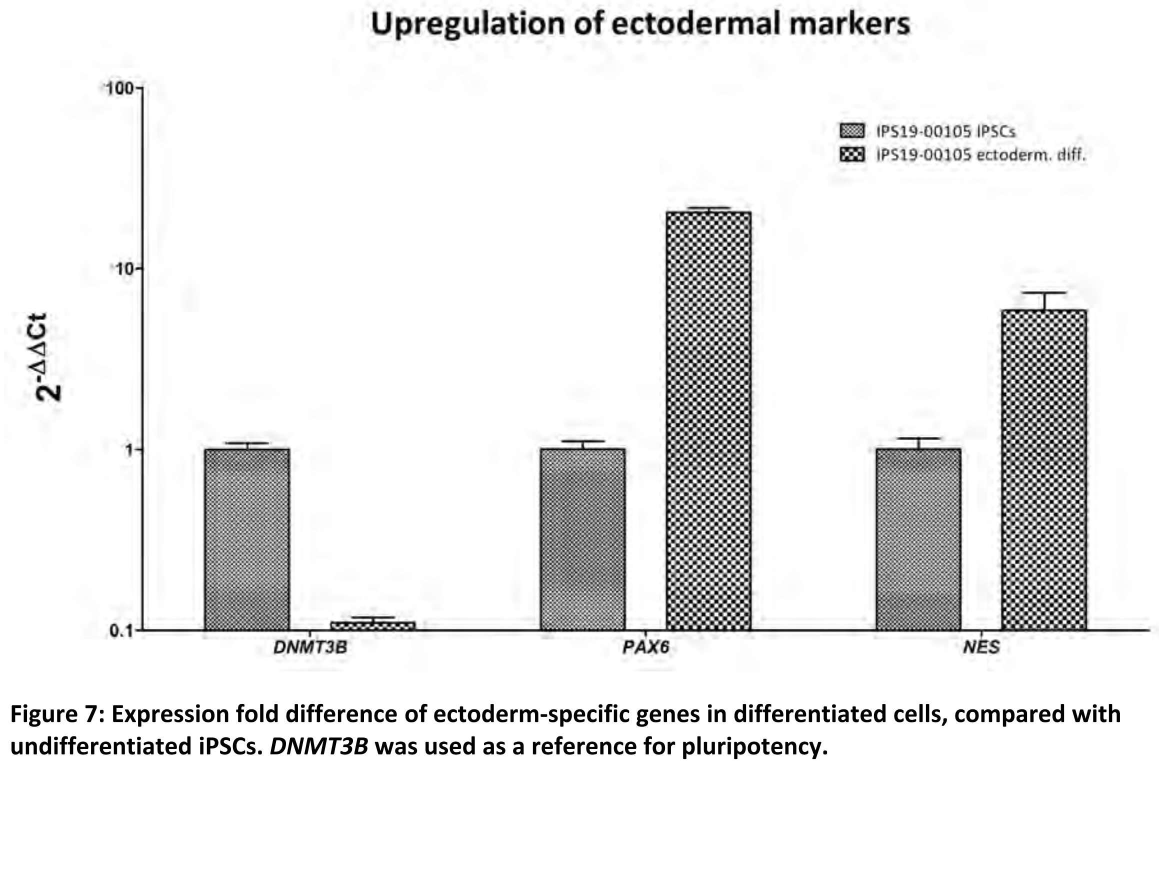

Figure 7: Expression fold difference of ectoderm-specific genes in differentiated cells, compared with undifferentiated iPSCs. *DNMT3B* was used as a reference for pluripotency.

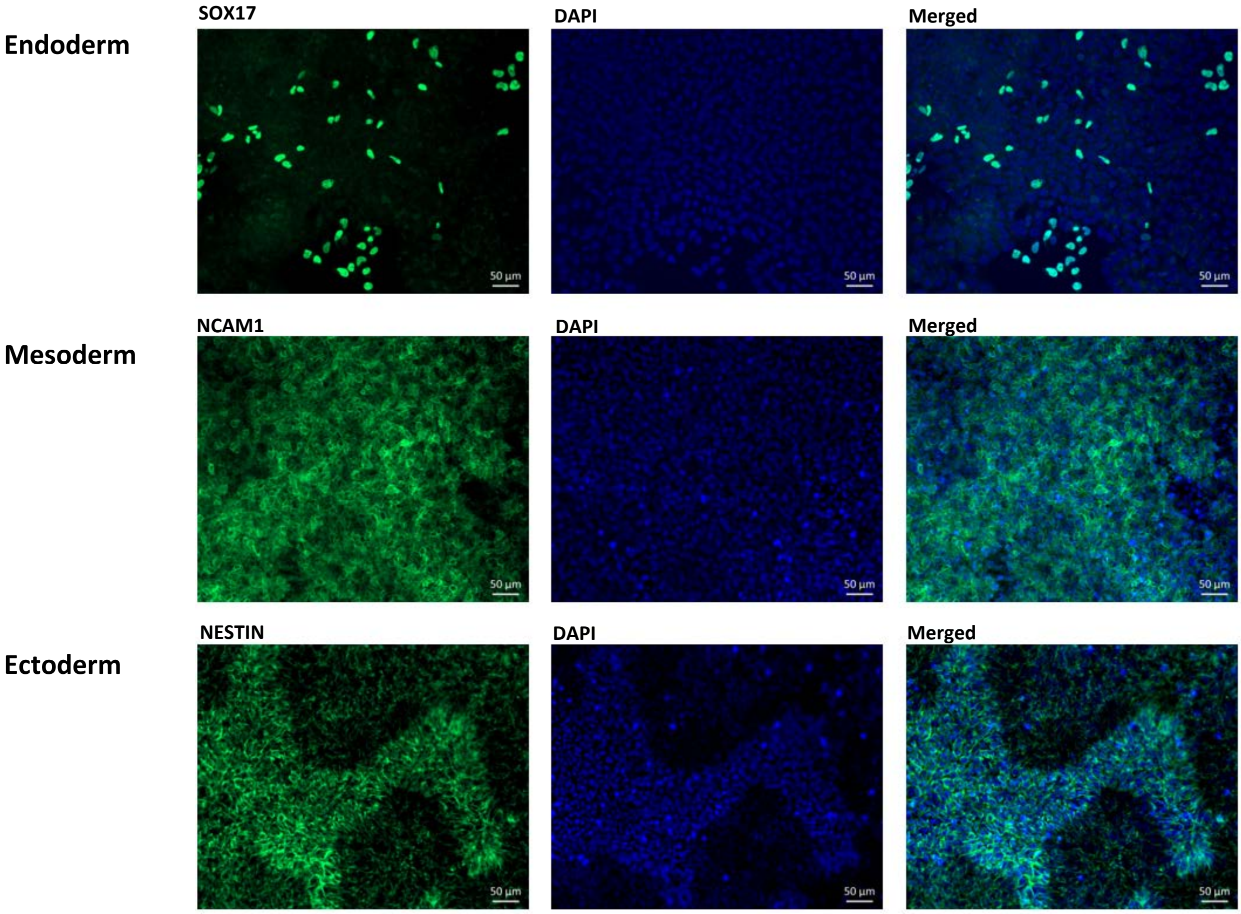

Figure 8: Immunofluorescence staining of differentiated cells showing positive signal of germlayer-specific markers.

Genetic analysis

DNA was isolated from three iPSC clones and the majority of recurrent chromosomal abnormalities reported in human embryonic stem cells and iPSCs was analysed.

Sample	Sex	Status
IPS19-00105 clone 1 p10	Female	Possibly Abnormal
IPS19-00105 clone 2 p10	Female	Possibly Abnormal
IPS19-00105 clone 3 p10	Female	Possibly Abnormal

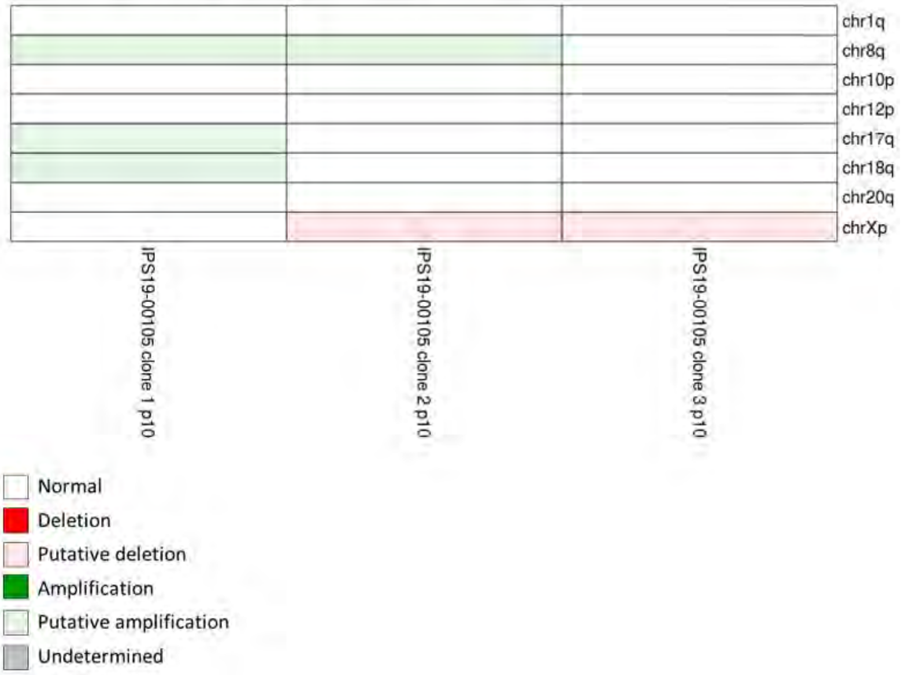

Figure 9: Summary of the genetic analysis

It is suggested to check IPS19-00105 clone 1, 2 and 3 at a later passage to assess whether there is indeed a mutant clone in the culture that expands over time.

More detailed results are on request.

Stem Cell Technology Center Genetica

Certificate of Analysis 2020

Invoice number: SCTC2019-00089

Name investigator: Christian Grimm
Cell line number: IPS19-00106
Project name: Zurich

Table 1: Information on the reprogrammed cell line

**Information cell line:**	
Product description	EBVs nucleofected with episomal vectors containing the genes OCT3/4, SOX2, KLF4, L-MYC, LIN28
Parental cell line	037365
Parental cell type	EBV immortalized B-lymphocytes
Diagnosis	AMD-S
Mutation	N/A*
Number of clones	3
Passage (P) of iPSCs reported at submission	P10
Culture medium	Essential 8 Flex medium
Culture coating	Matrigel
Feeders during reprogramming	Mouse Embryonic Fibroblasts (MEFs)
Passage method	0.5 mM EDTA
Protocols in Q-portal	046588; 046591

Table 2: Information on the characterization of the reprogrammed cell line

**Test description:**	**Test method:**	**Test specification:**	**Result:**
Activation of stem cell markers	qPCR	Upregulation of *SOX2, LIN28, NANOG, DNMT3B* compared with EBVs	Pass
Expression of stem cell markers	Immunocytochemistry	Expression of OCT4, NANOG, SSEA4, TRA-1-81	Pass
Mycoplasma	PCR	Negative	Pass
Three lineage differentiation	Differentiation assay	Upregulation of germlayer-specific genes	Pass
hPSC genetic analysis	qPCR	Detection of recurrent chromosomal abnormalities	See results in last page

*N/A: Not applicable

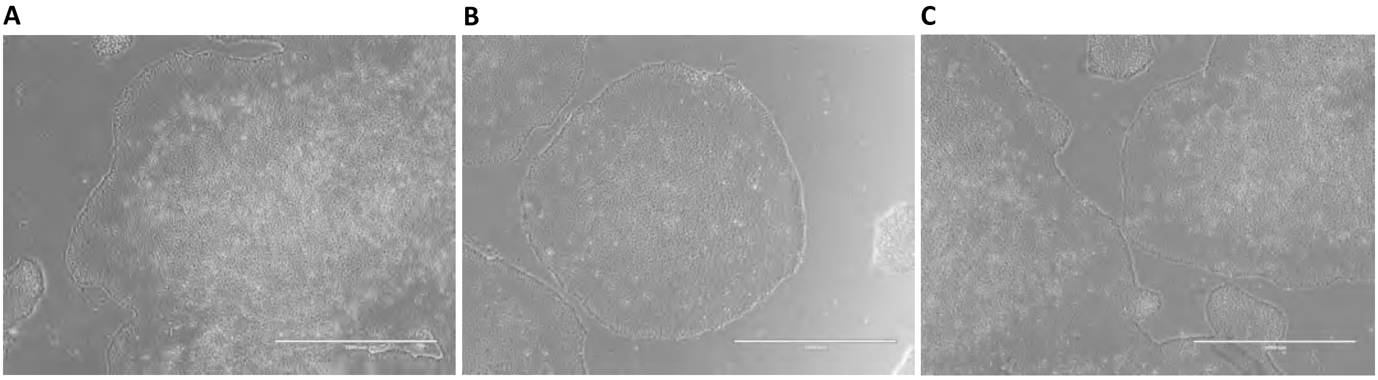

Figure 1: Cells prior to freezing. A - C, clone 1, clone 2 and clone 3, respectively at P10. Scale bar = 1000 µm.

Activation of stem cell markers

All clones were assessed for activation of stem cell markers before freezing. RNA was isolated and gene expression was assessed by quantitative reverse transcription PCR. Ct values were normalized with the housekeeping gene GUSB (set at 1).

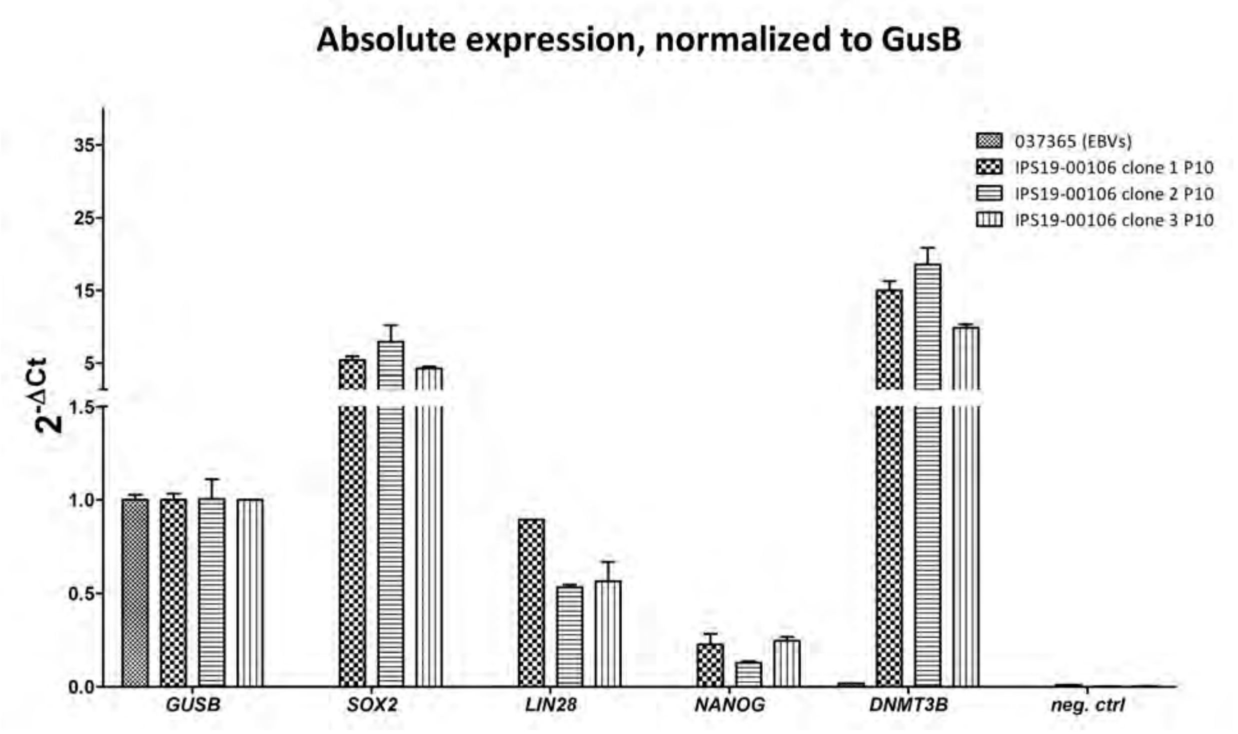

Figure 2: Gene expression of three iPSC clones compared with the parental EBVs (ΔCt).

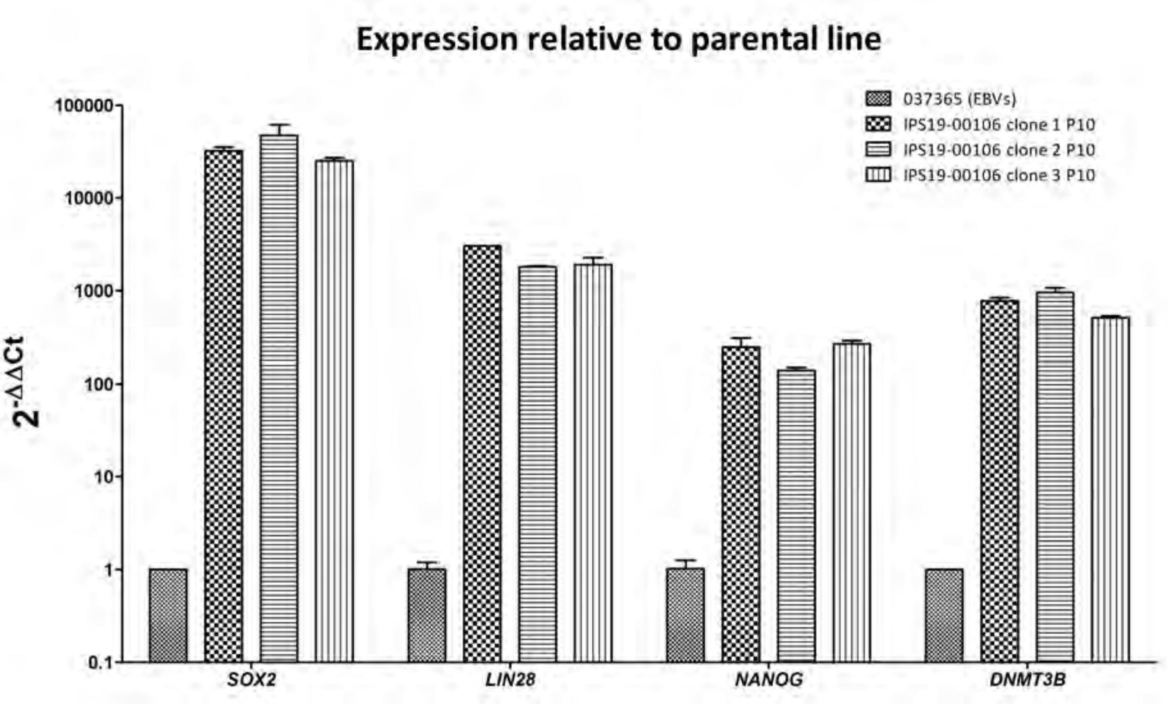

Figure 3: Pluripotency gene upregulation after reprogramming (ΔΔCt). The expression fold difference of the iPSCs is relative to the parental EBVs.

Expression of stem cell markers

Undifferentiated iPSC clones were stained for the nuclear markers NANOG and OCT4 and surface antigens SSEA4 and TRA-1-81. All markers are expressed in human pluripotent stem cells.

A. IPS19-00106 clone 1

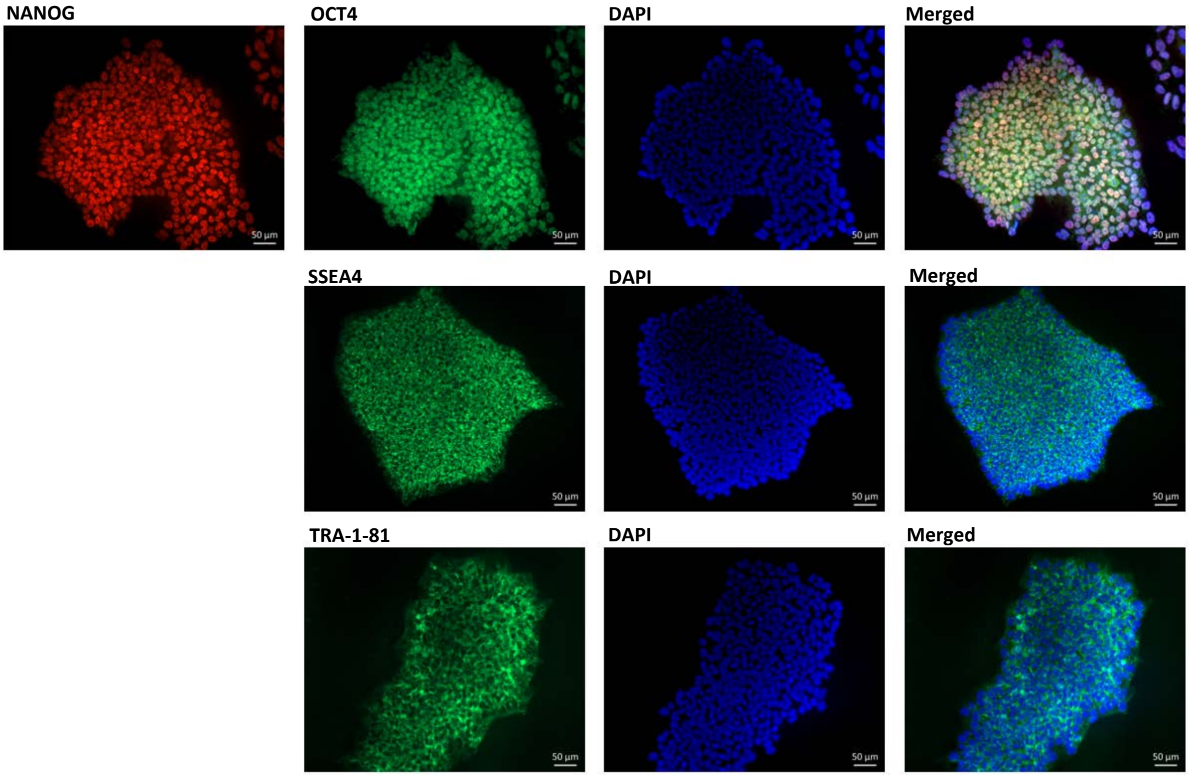

B. IPS19-00106 clone 2

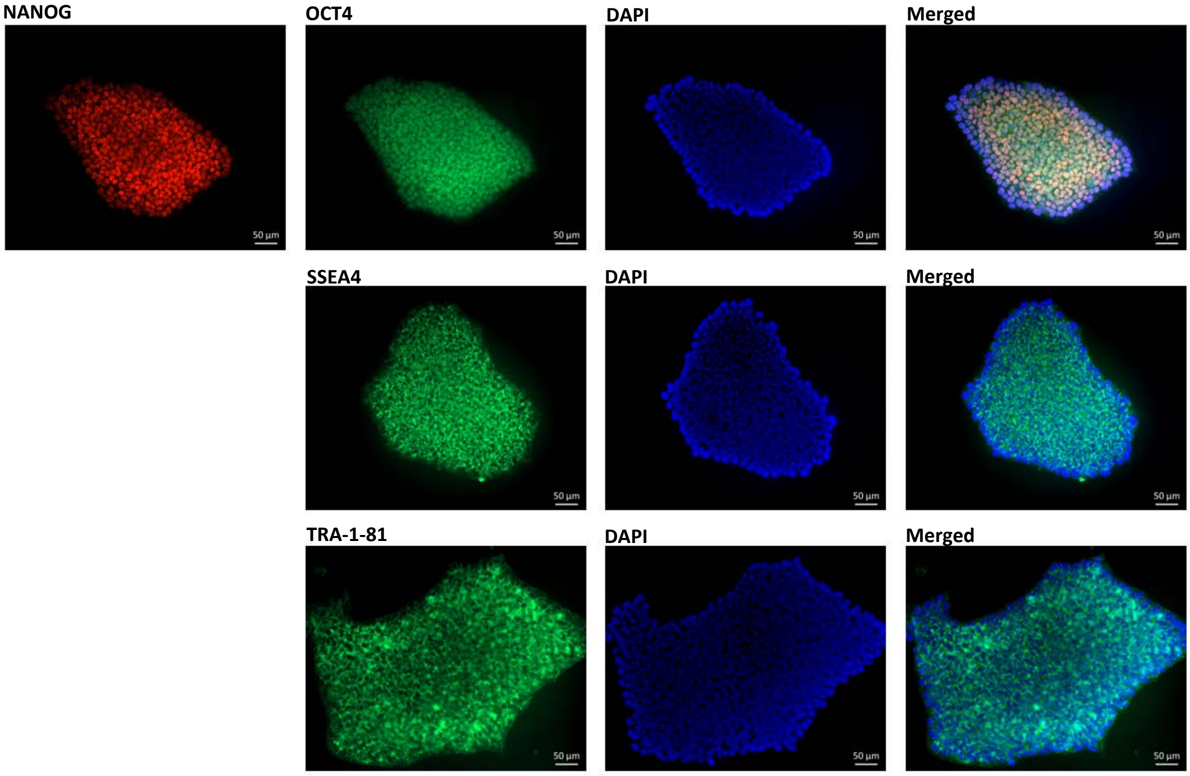

C. IPS19-00106 clone 3

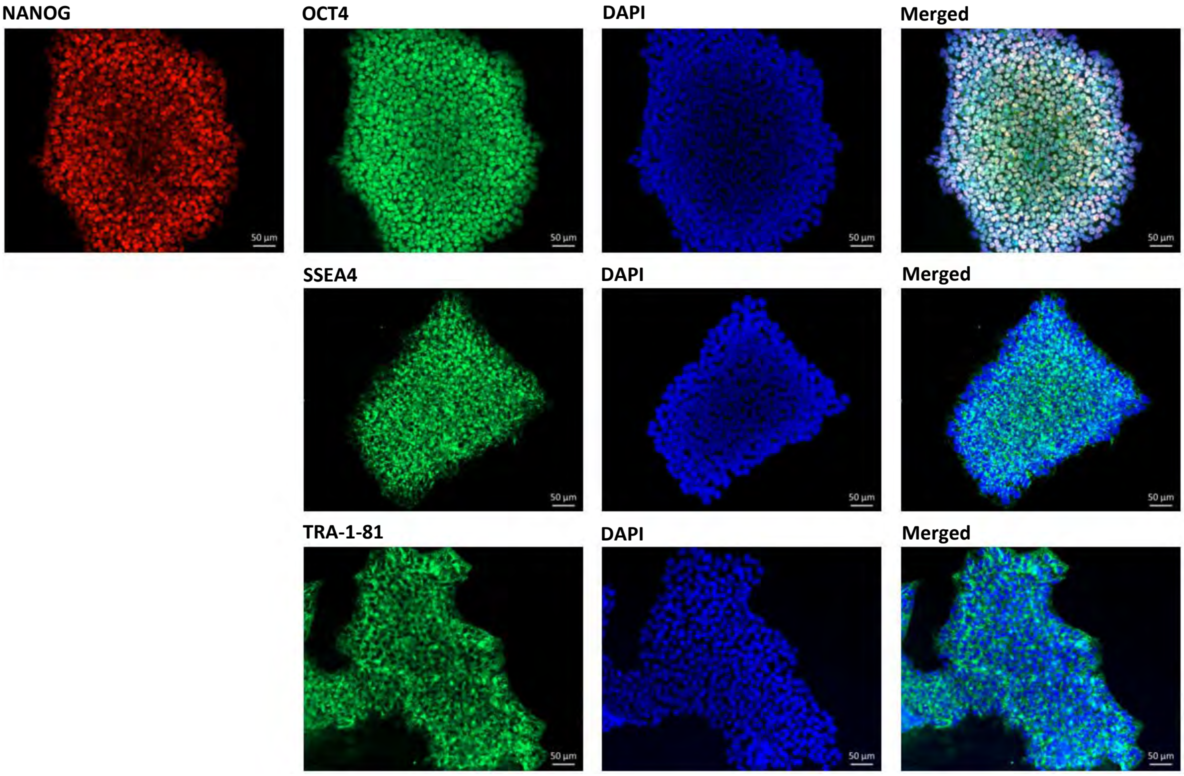

Figure 4: Immunofluorescence staining of the iPSC clones with pluripotency markers.

Three germ layer differentiation

IPS19-00106 clone 1 was differentiated into the endodermal, mesodermal and ectodermal germ layers. RNA was isolated and gene expression was checked by qPCR. Ct values are normalized with the housekeeping gene GUSB (set at 1). For each lineage two genes were assessed (Table 3). The differentiated cells were also stained for lineage-specific markers (Table 4).

Table 3: qPCR markers for three lineage differentiation

Lineage	Marker
Endoderm	FOXA2, SOX17
Mesoderm	Brachyury, HAND1
Ectoderm	PAX6, NCAM1

Table 4: ICC markers for three lineage differentiation

Lineage	Marker
Endoderm	SOX17
Mesoderm	NCAM1
Ectoderm	NESTIN

Endoderm

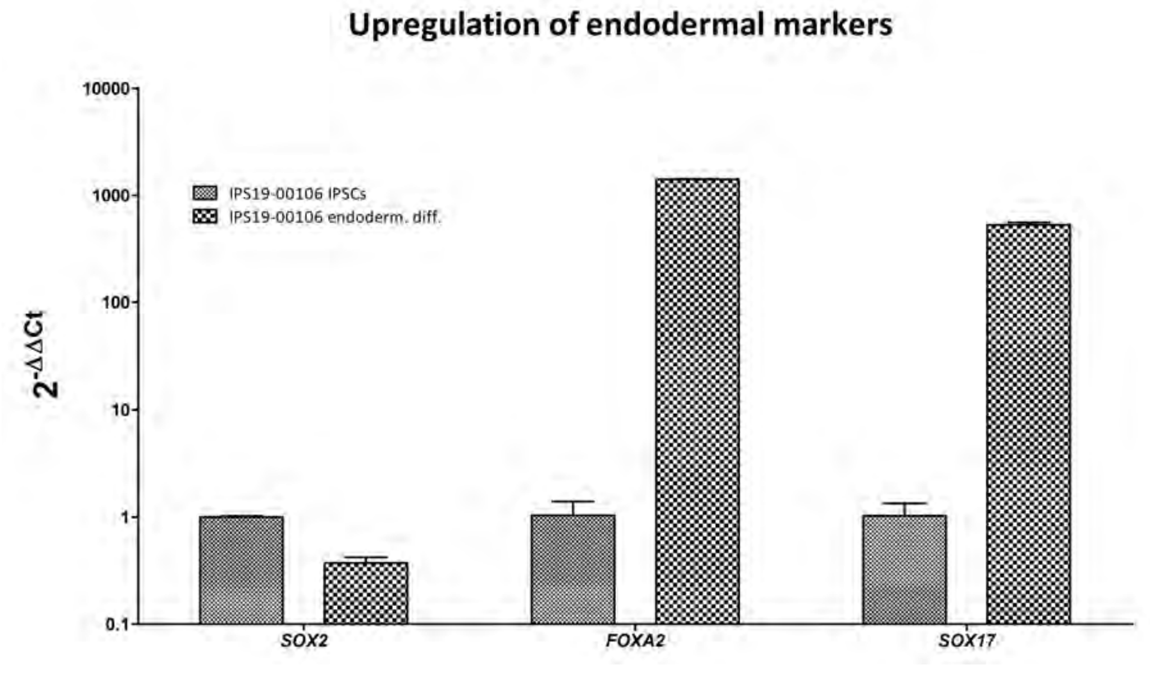

Figure 5: Expression fold difference of endoderm-specific genes in differentiated cells, compared with undifferentiated iPSCs. *SOX2* was used as a reference for pluripotency.

Mesoderm

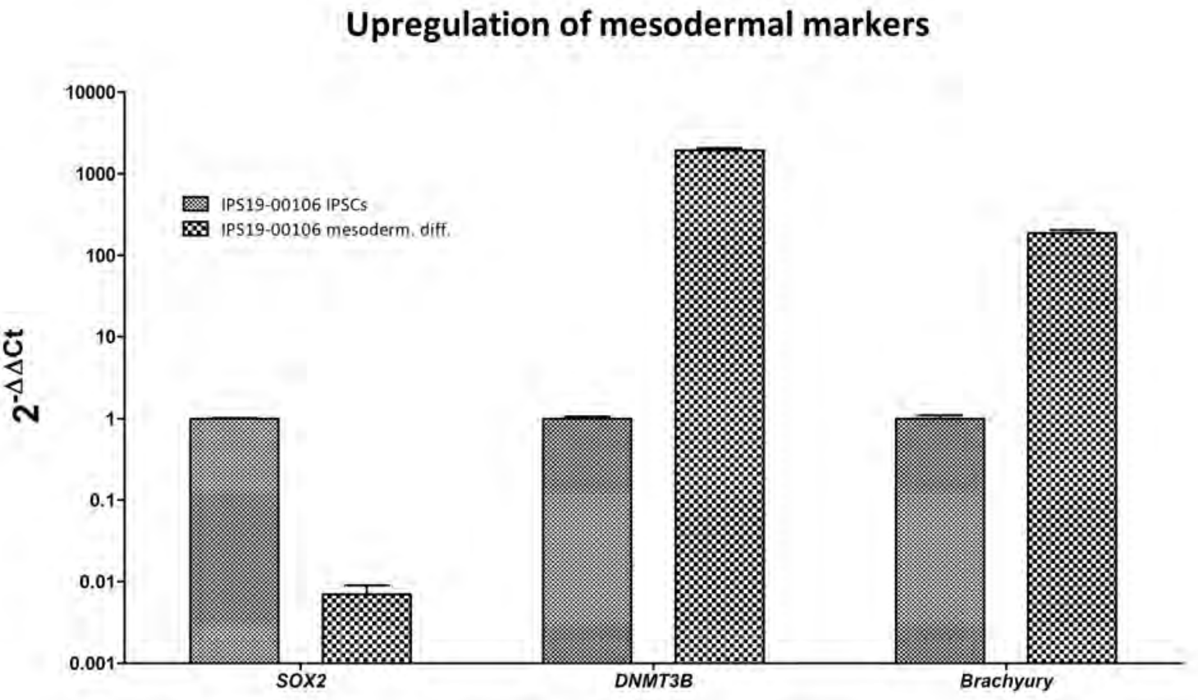

Figure 6: Expression fold difference of mesoderm-specific genes in differentiated cells, compared with undifferentiated iPSCs. *SOX2* was used as a reference for pluripotency.

Ectoderm

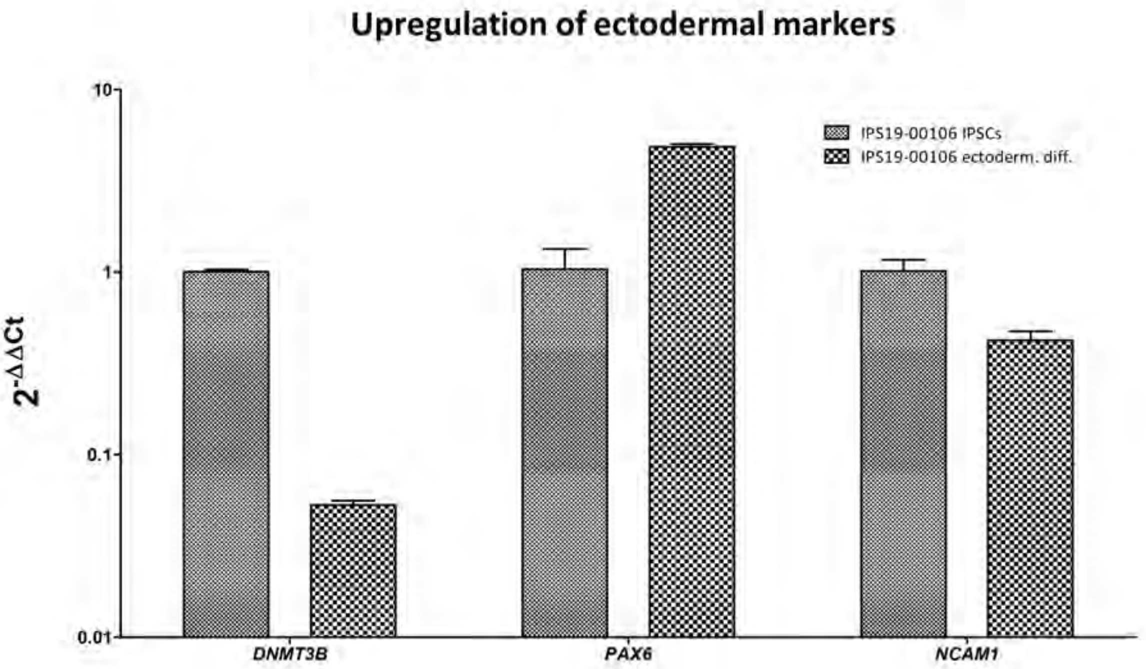

Figure 7: Expression fold difference of ectoderm-specific genes in differentiated cells, compared with undifferentiated iPSCs. *DNMT3B* was used as a reference for pluripotency.

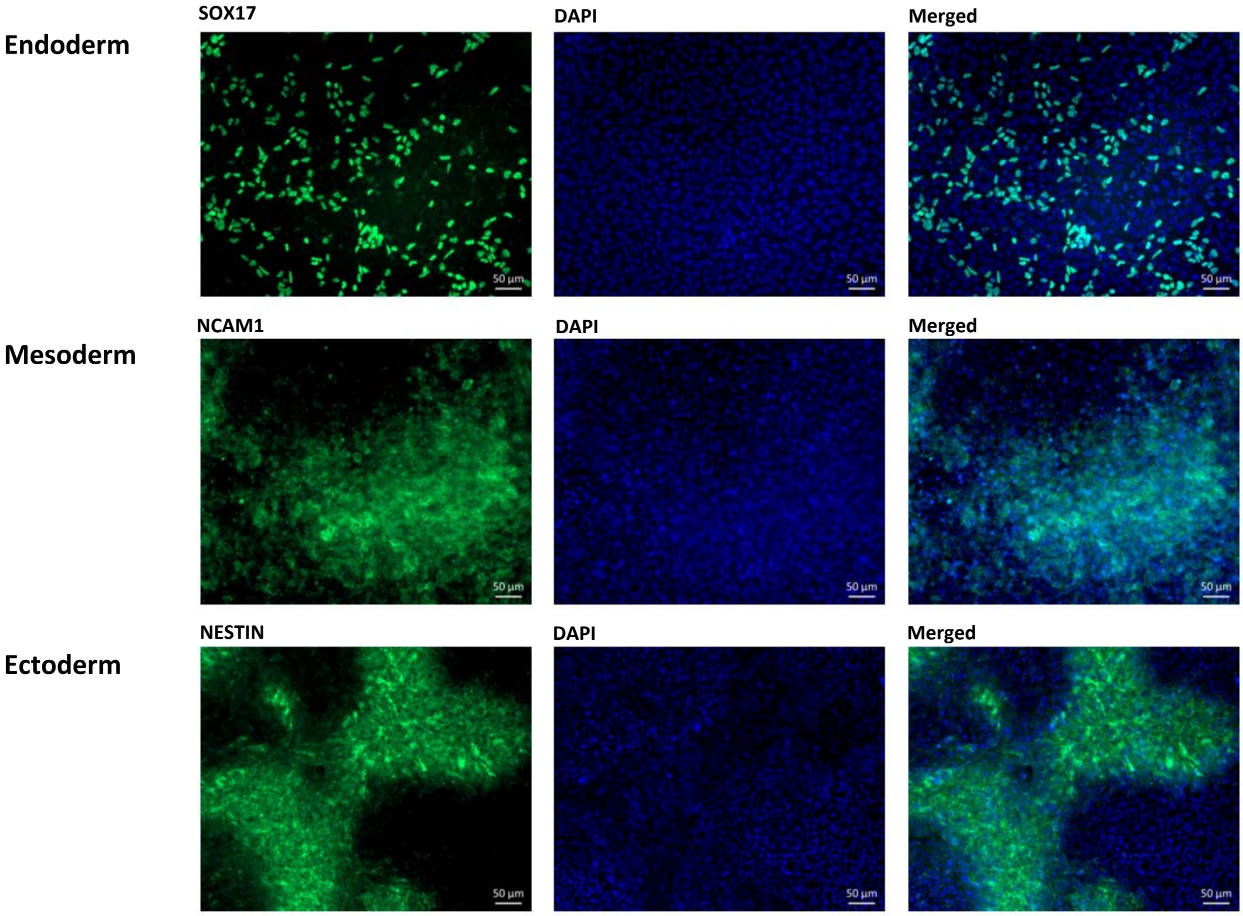

Figure 8: Immunofluorescence staining of differentiated cells showing positive signal of germlayer-specific markers.

Genetic analysis

DNA was isolated from three iPSC clones and the majority of recurrent chromosomal abnormalities reported in human embryonic stem cells and iPSCs was analysed.

Sample	Sex	Status
IPS19-00106 clone 1 P10	Male	Possibly Abnormal
IPS19-00106 clone 2 P10	Male	Normal (Sex Difference)
**IPS19-00106 clone 3 P10**	**Male**	**Abnormal**

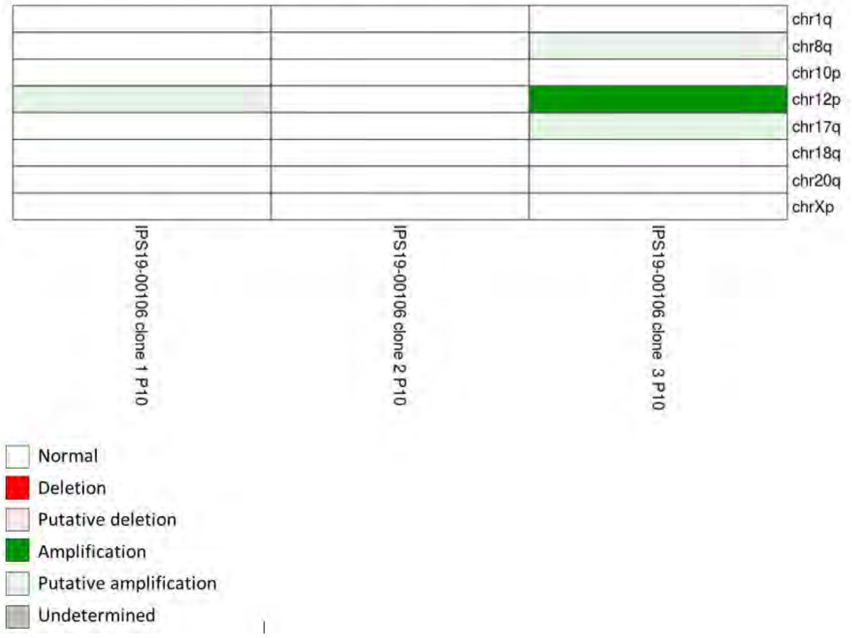

Figure 9: Summary of the genetic analysis

For further experiments it is suggested to use IPS19-00106 clone 2. It is suggested to check IPS19-00106 clone 1 and 3 at a later passage to assess whether there is indeed a mutant clone in the culture that expands over time.

More detailed results are on request.

Certificate of Analysis 2020

Invoice number: SCTC2019-00085

Name investigator: Christian Grimm
Cell line number: IPS19-00096
Project name: Zürich

Table 1: Information on the reprogrammed cell line

**Information cell line:**	
Product description	EBVs nucleofected with episomal vectors containing the genes OCT3/4, SOX2, KLF4, L-MYC, LIN28
Parental cell line	039812
Parental cell type	EBV immortalized B-lymphocytes
Diagnosis	AMD-D
Mutation	N/A*
Number of clones	3
Passage (P) of iPSCs reported at submission	P10
Culture medium	Essential 8 Flex medium
Culture coating	Matrigel
Feeders during reprogramming	Mouse Embryonic Fibroblasts (MEFs)
Passage method	0.5 mM EDTA
Protocols in Q-portal	046588; 046591

Table 2: Information on the characterization of the reprogrammed cell line

**Test description:**	**Test method:**	**Test specification:**	**Result:**
Activation of stem cell markers	qPCR	Upregulation of *SOX2, LIN28, NANOG, DNMT3B* compared with EBVs	Pass
Expression of stem cell markers	Immunocytochemistry	Expression of OCT4, NANOG, SSEA4, TRA-1-81	Pass
Mycoplasma	PCR	Negative	Pass
Three lineage differentiation	Differentiation assay	Upregulation of germlayer-specific genes	Pass
hPSC genetic analysis	qPCR	Detection of recurrent chromosomal abnormalities	See results in last page

*N/A: Not applicable

A **B** **C**

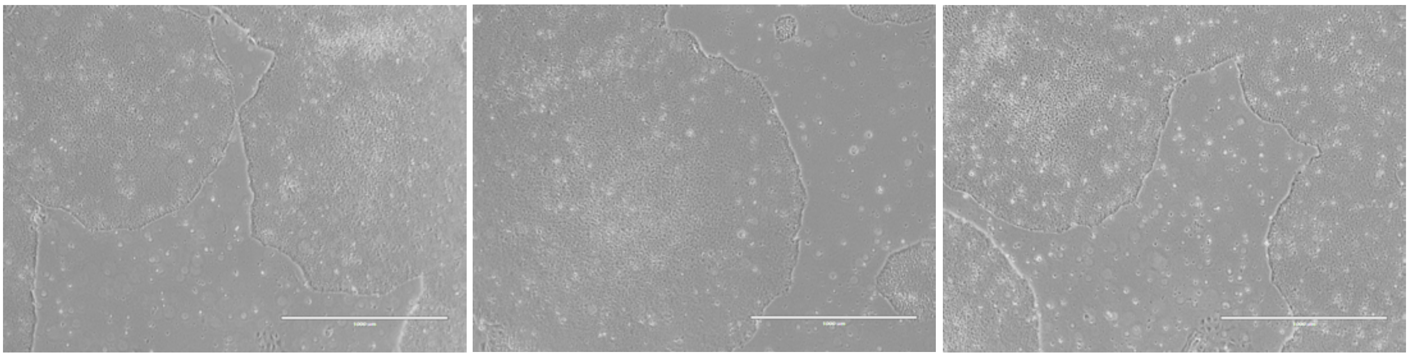

Figure 1: Cells prior to freezing. A - C, clone 1, clone 2 and clone 3, respectively at P10. Scale bar = 1000 µm.

Activation of stem cell markers

All clones were assessed for activation of stem cell markers before freezing. RNA was isolated and gene expression was assessed by quantitative reverse transcription PCR. Ct values were normalized with the housekeeping gene GUSB (set at 1).

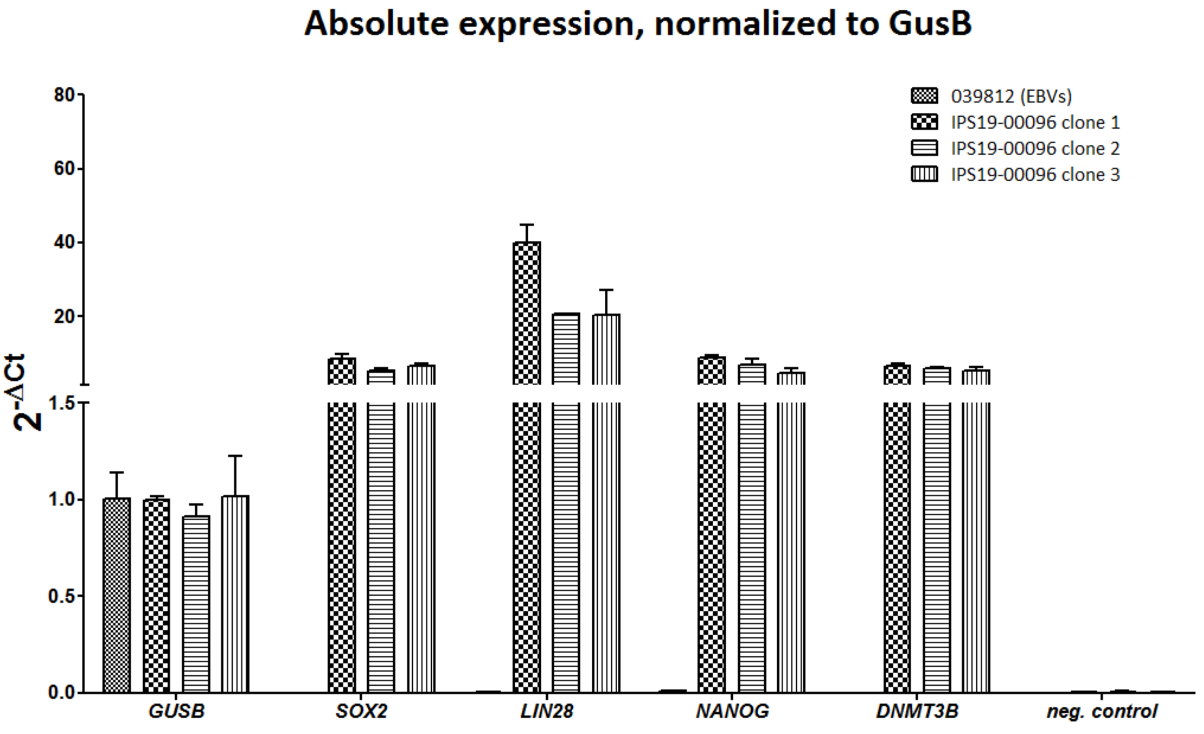

Figure 2: Gene expression of three iPSC clones compared with the parental EBVs (ΔCt).

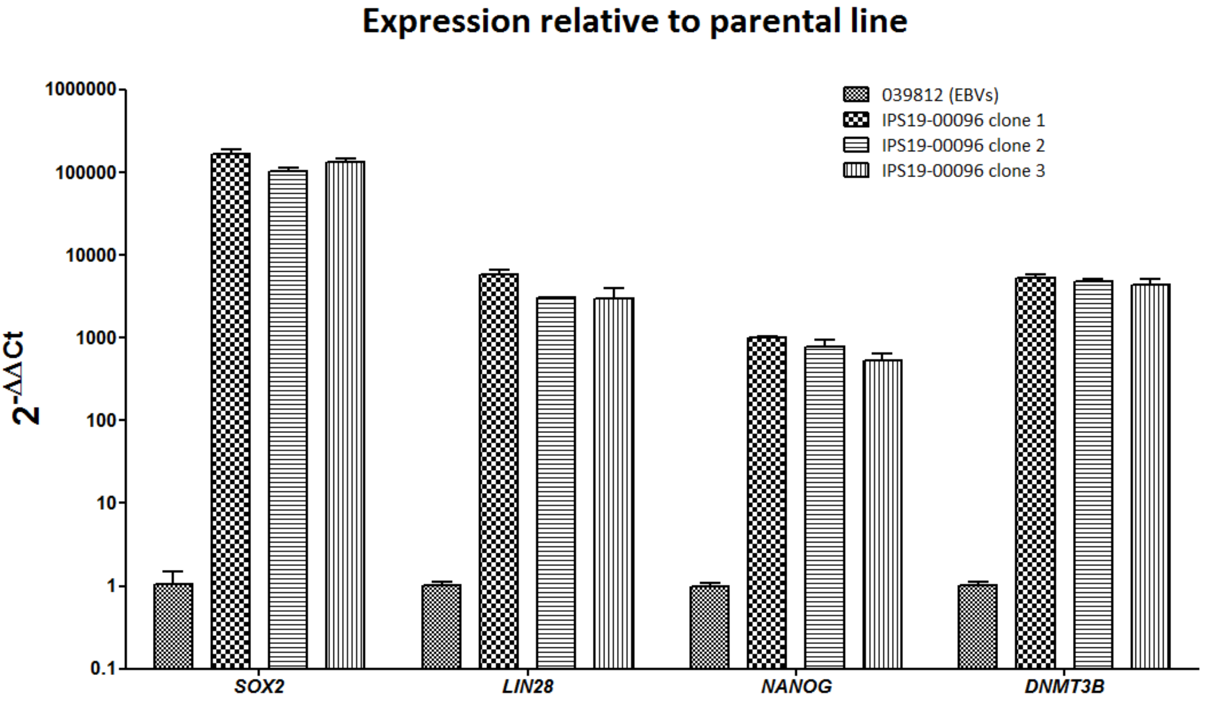

Figure 3: Pluripotency gene upregulation after reprogramming (ΔΔCt). The expression fold difference of the iPSCs is relative to the parental EBVs.

Expression of stem cell markers

Undifferentiated iPSC clones were stained for the nuclear markers NANOG and OCT4 and surface antigens SSEA4 and TRA-1-81. All markers are expressed in human pluripotent stem cells.

A. IPS19-00096 clone 1

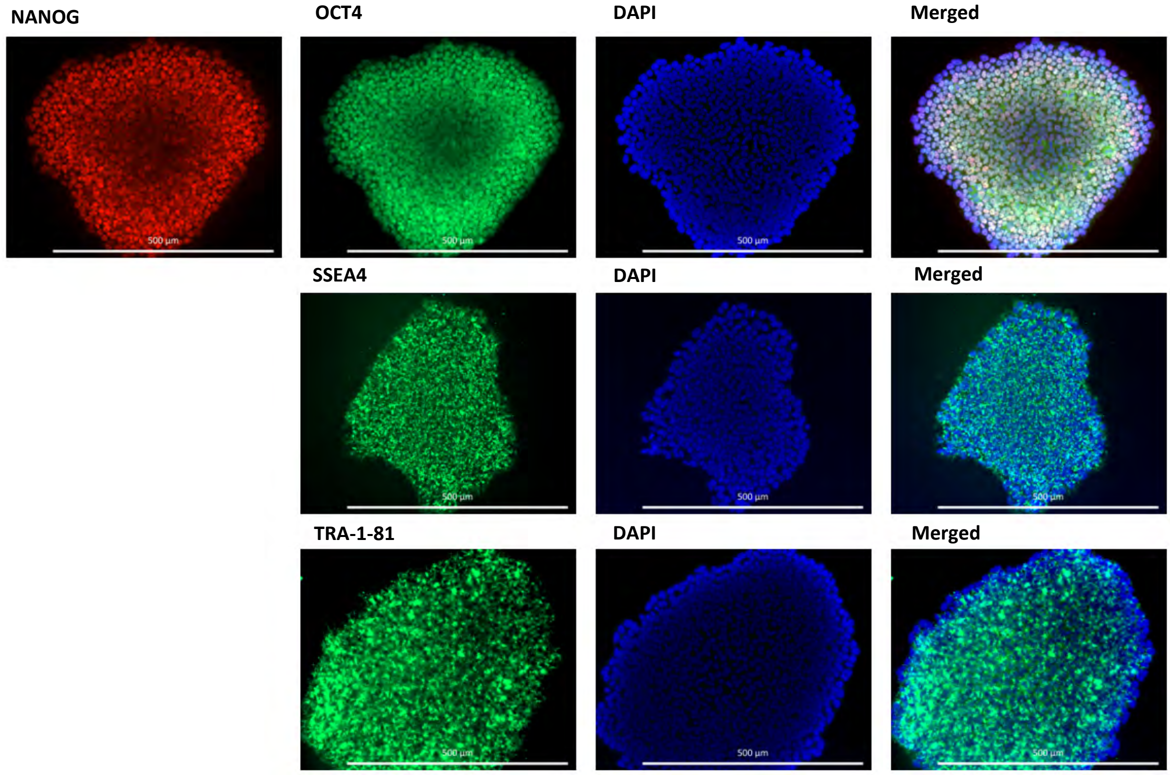

B. IPS19-00096 clone 2

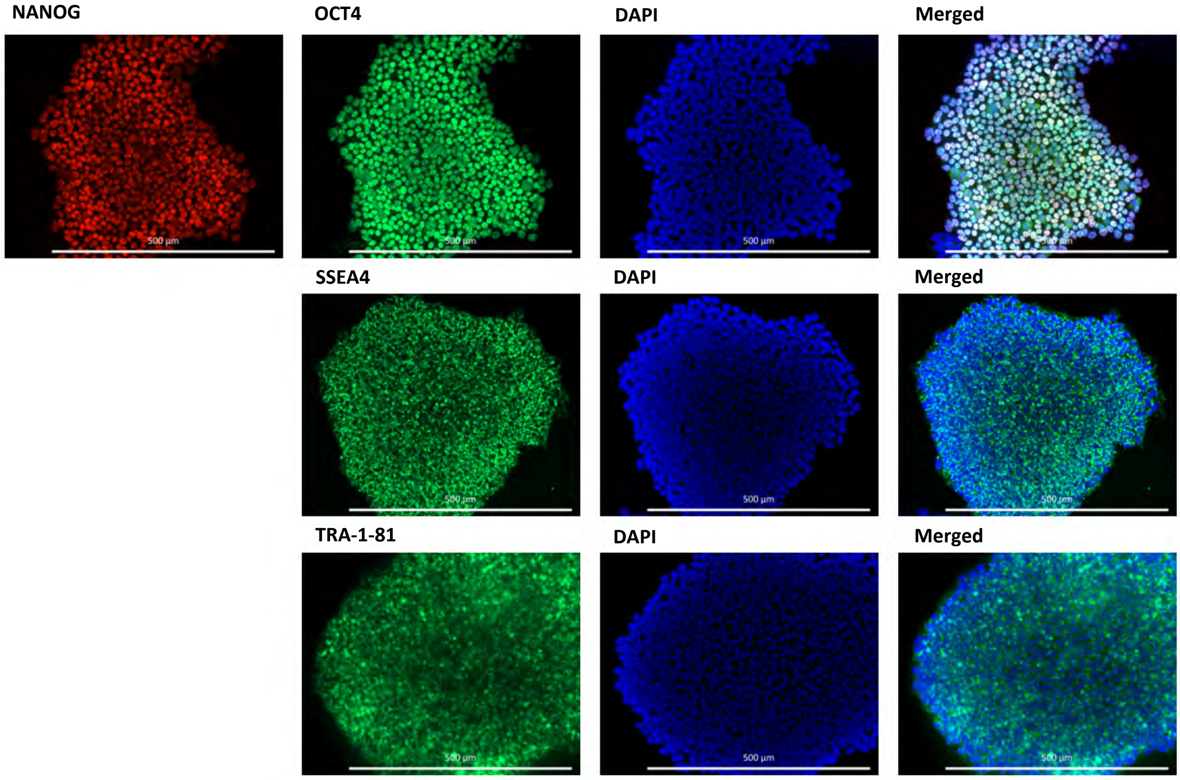

C. IPS19-00096 clone 3

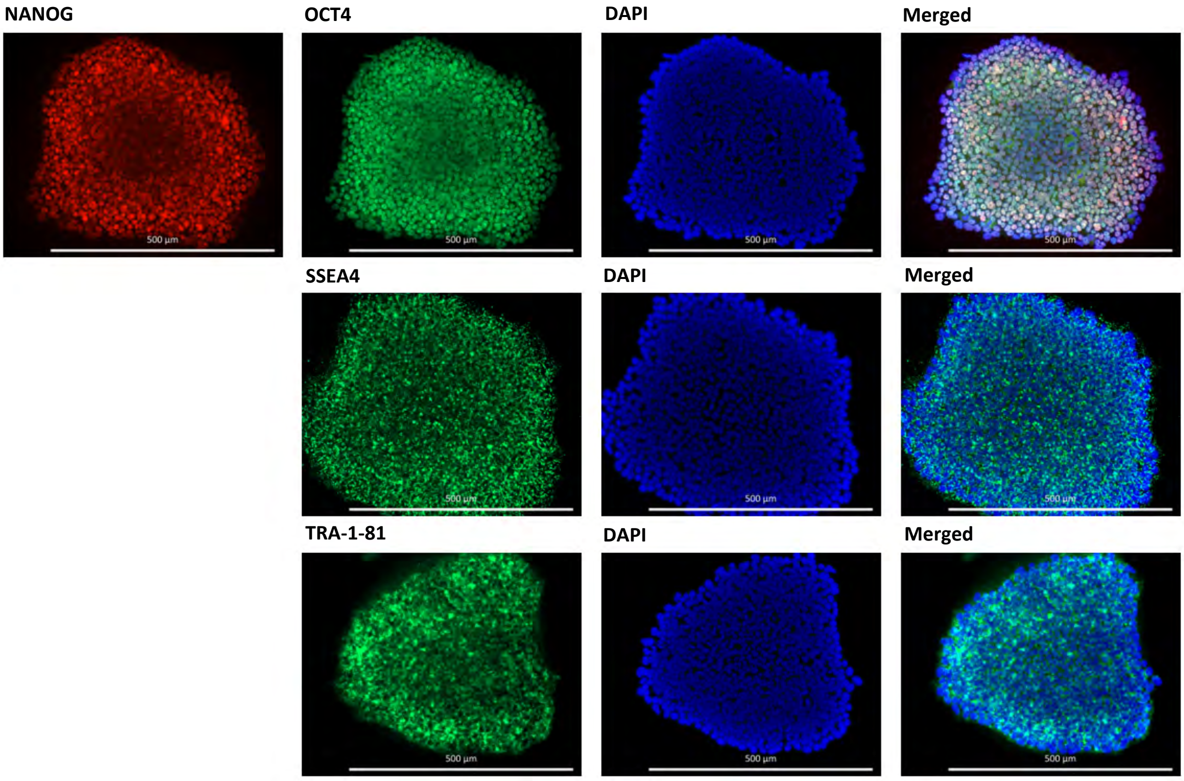

Figure 4: Immunofluorescence staining of the iPSC clones with pluripotency markers.

Three germ layer differentiation

IPS19-00096 clone 1 was differentiated into the endodermal, mesodermal and ectodermal germ layers. RNA was isolated and gene expression was checked by qPCR. Ct values are normalized with the housekeeping gene GUSB (set at 1). For each lineage two genes were assessed (Table 3). The differentiated cells were also stained for lineage-specific markers (Table 4).

Table 3: qPCR markers for three lineage differentiation

Lineage	Marker
Endoderm	FOXA2, SOX17
Mesoderm	Brachyury, HAND1
Ectoderm	PAX6, NES

Table 4: ICC markers for three lineage differentiation

Lineage	Marker
Endoderm	SOX17
Mesoderm	NCAM1
Ectoderm	NESTIN

Endoderm

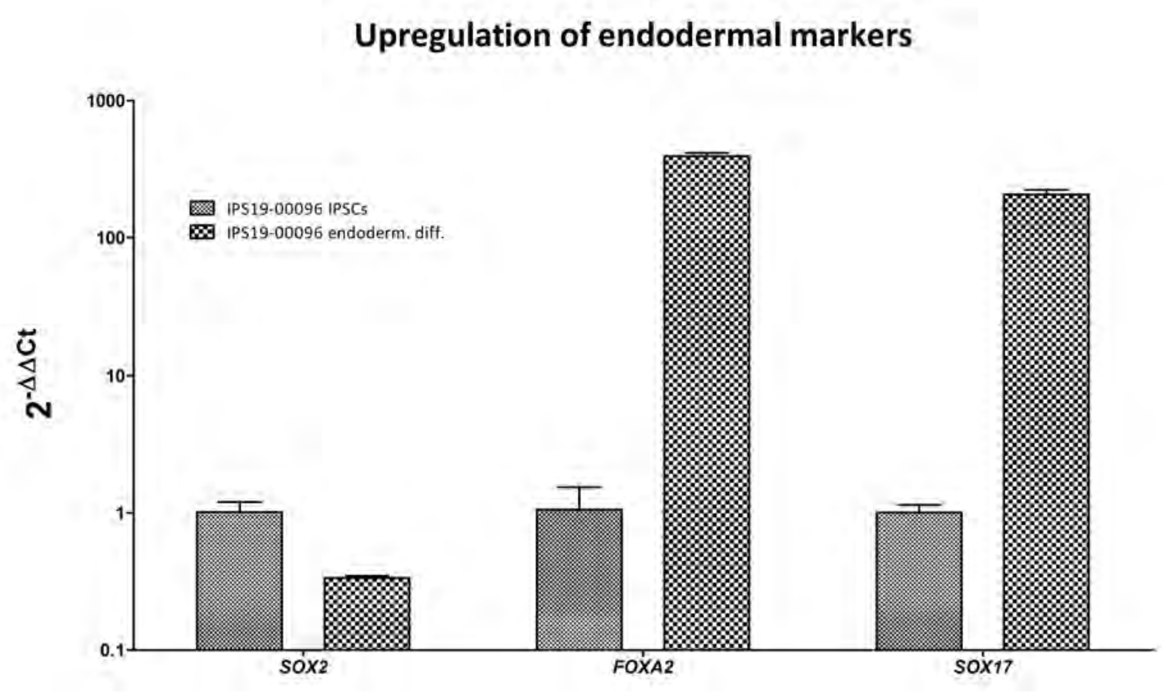

Figure 5: Expression fold difference of endoderm-specific genes in differentiated cells, compared with undifferentiated iPSCs. *SOX2* was used as a reference for pluripotency.

Mesoderm

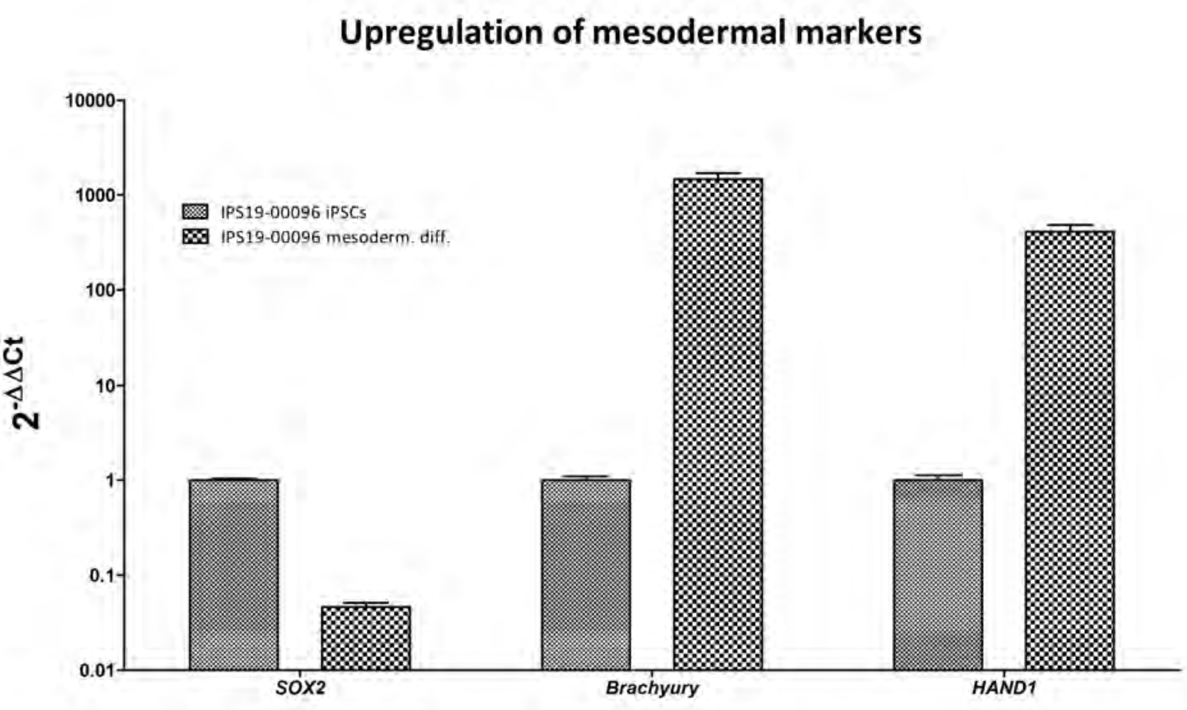

Figure 6: Expression fold difference of mesoderm-specific genes in differentiated cells, compared with undifferentiated iPSCs. *SOX2* was used as a reference for pluripotency.

Ectoderm

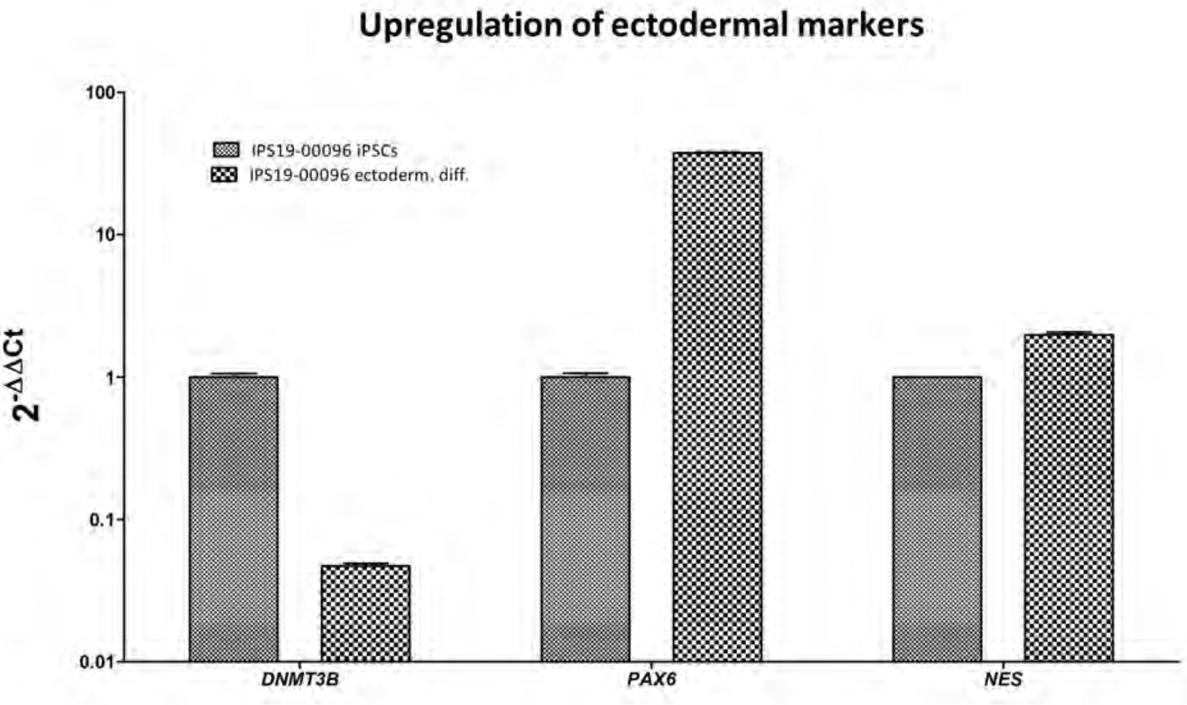

Figure 7: Expression fold difference of ectoderm-specific genes in differentiated cells, compared with undifferentiated iPSCs. *DNMT3B* was used as a reference for pluripotency.

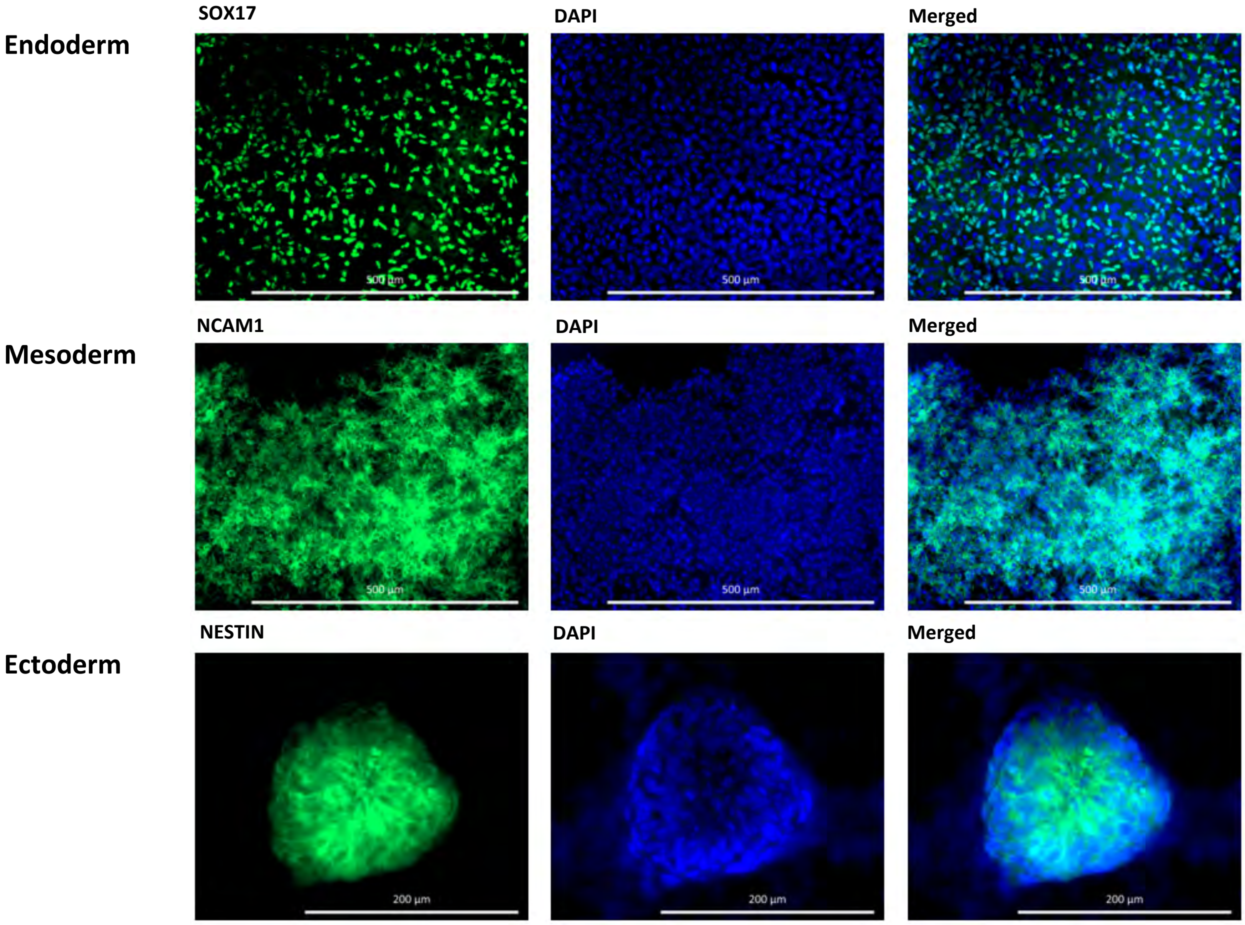

Figure 8: Immunofluorescence staining of differentiated cells showing positive signal of germlayer-specific markers.

Genetic analysis

DNA was isolated from three iPSC clones and the majority of recurrent chromosomal abnormalities reported in human embryonic stem cells and iPSCs was analysed.

Sample	Sex	Status
**IPS19-00096 clone 1 P10**	**Female**	**Abnormal**
IPS19-00096 clone 2 P10	Female	Normal
IPS19-00096 clone 3 P10	Female	Normal

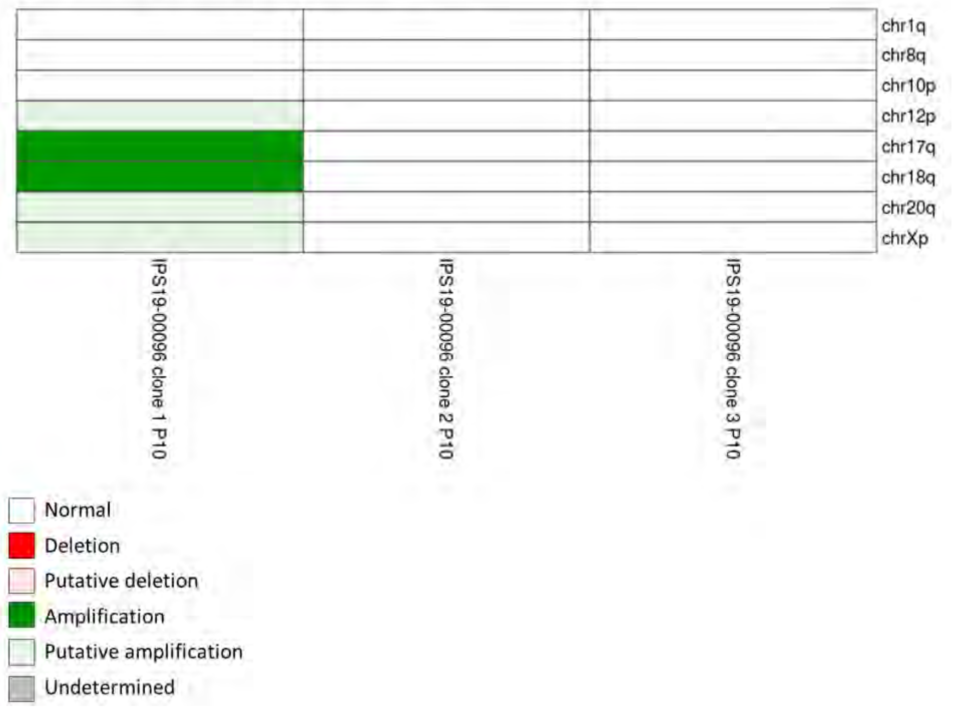

Figure 9: Summary of the genetic analysis

For further experiments it is suggested to use IPS19-00096 clone 2 and 3. It is suggested to check IPS19-00096 clone 1 at a later passage to assess whether there is indeed a mutant clone in the culture that expands over time.

More detailed results are on request.

Stem Cell Technology Center Genetica

Certificate of Analysis 2020

Invoice number: SCTC2019-00087

Name investigator: Christian Grimm
Cell line number: IPS19-00097
Project name: Zurich

Table 1: Information on the reprogrammed cell line

Information cell line:	
Product description	EBVs nucleofected with episomal vectors containing the genes OCT3/4, SOX2, KLF4, L-MYC, LIN28
Parental cell line	HEP09-01068
Parental cell type	EBV immortalized B-lymphocytes
Diagnosis	AMD
Mutation	N/A*
Number of clones	3
Passage (P) of iPSCs reported at submission	P10
Culture medium	Essential 8 Flex medium
Culture coating	Matrigel
Feeders during reprogramming	Mouse Embryonic Fibroblasts (MEFs)
Passage method	0.5 mM EDTA
Protocols in Q-portal	046588; 046591

Table 2: Information on the characterization of the reprogrammed cell line

Test description:	Test method:	Test specification:	Result:
Activation of stem cell markers	qPCR	Upregulation of *SOX2, LIN28, NANOG, DNMT3B* compared with EBVs	Pass
Expression of stem cell markers	Immunocytochemistry	Expression of OCT4, NANOG, SSEA4, TRA-1-81	Pass
Mycoplasma	PCR	Negative	Pass
Three lineage differentiation	Differentiation assay	Upregulation of germlayer-specific genes	Pass
hPSC genetic analysis	qPCR	Detection of recurrent chromosomal abnormalities	See results in last page

*N/A: Not applicable

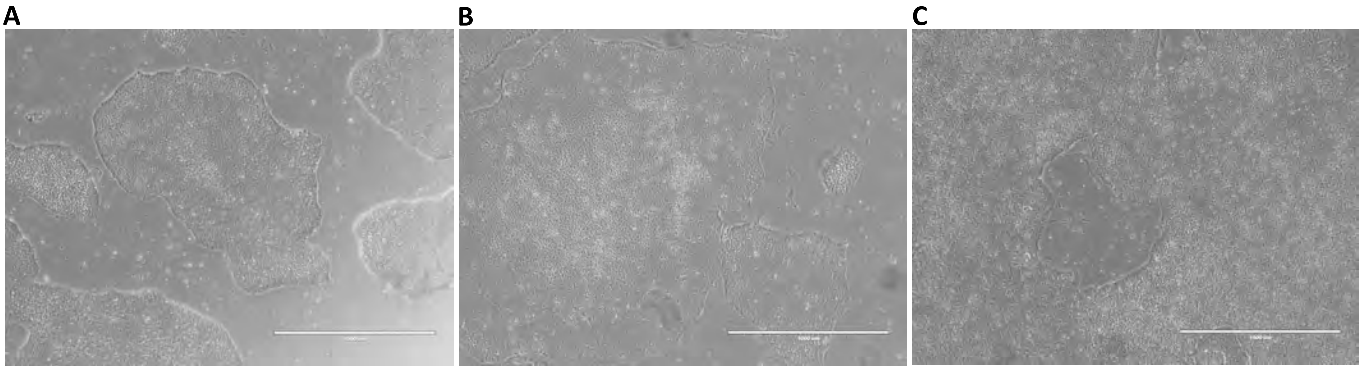

Figure 1: Cells prior to freezing. A - C, clone 1, clone 2 and clone 3, respectively at P10. Scale bar = 1000 µm.

Activation of stem cell markers

All clones were assessed for activation of stem cell markers before freezing. RNA was isolated and gene expression was assessed by quantitative reverse transcription PCR. Ct values were normalized with the housekeeping gene GUSB (set at 1).

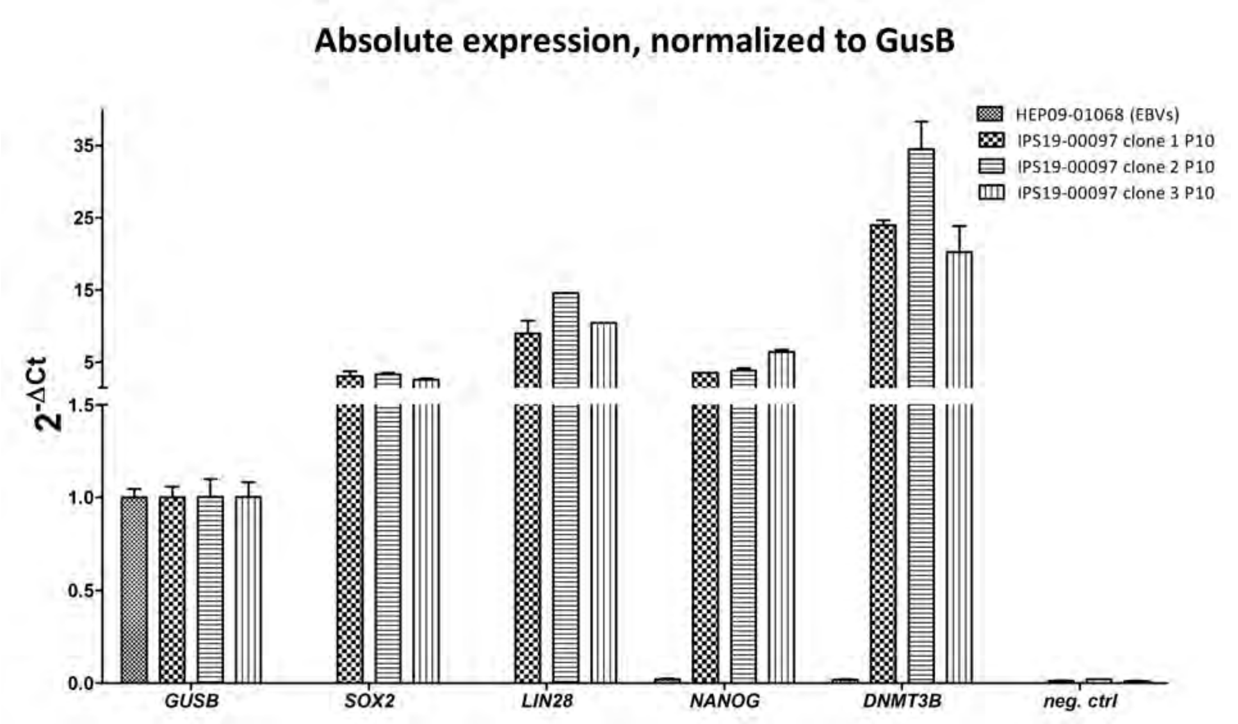

Figure 2: Gene expression of three iPSC clones compared with the parental EBVs (ΔCt).

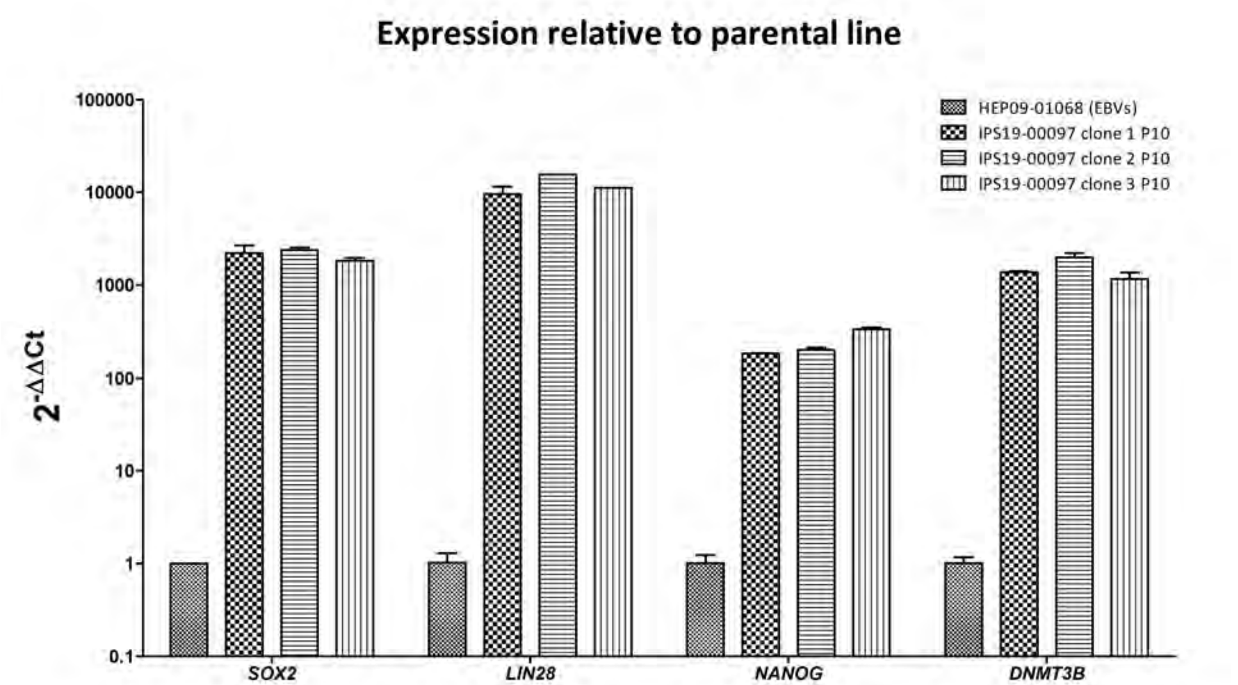

Figure 3: Pluripotency gene upregulation after reprogramming (ΔΔCt). The expression fold difference of the iPSCs is relative to the parental EBVs.

Expression of stem cell markers

Undifferentiated iPSC clones were stained for the nuclear markers NANOG and OCT4 and surface antigens SSEA4 and TRA-1-81. All markers are expressed in human pluripotent stem cells.

A. IPS19-00097 clone 1

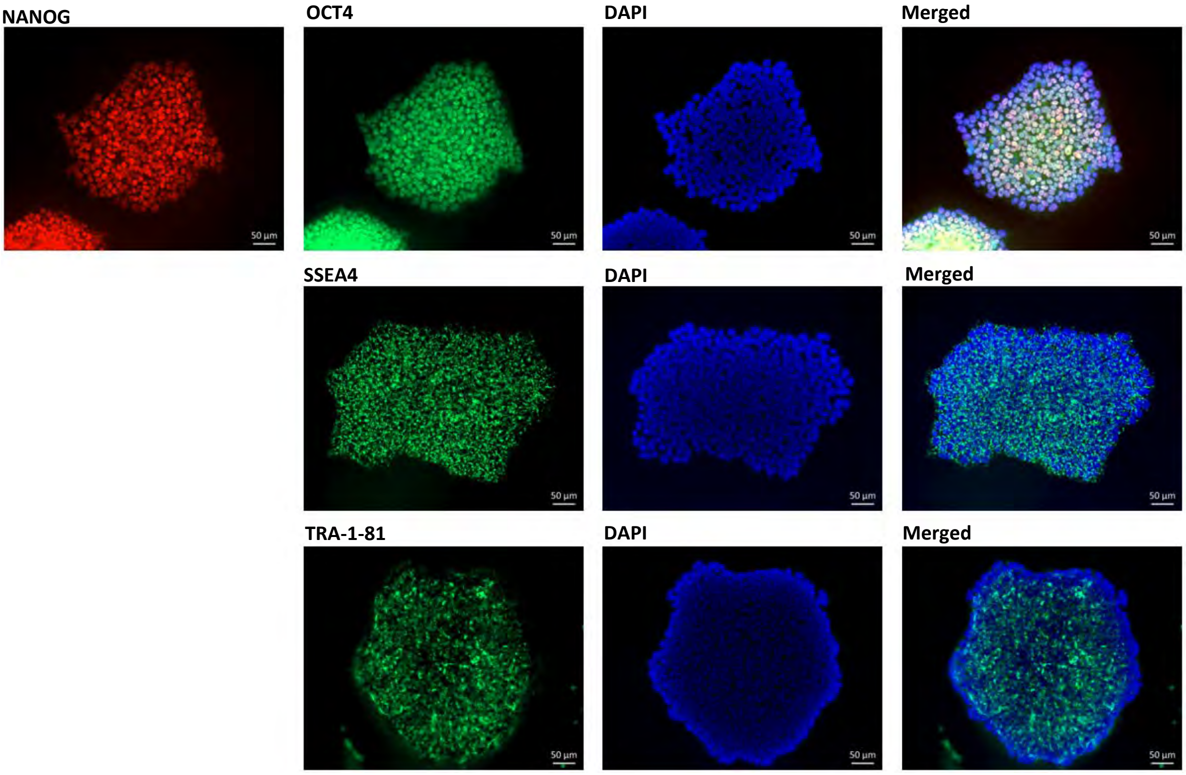

B. IPS19-00097 clone 2

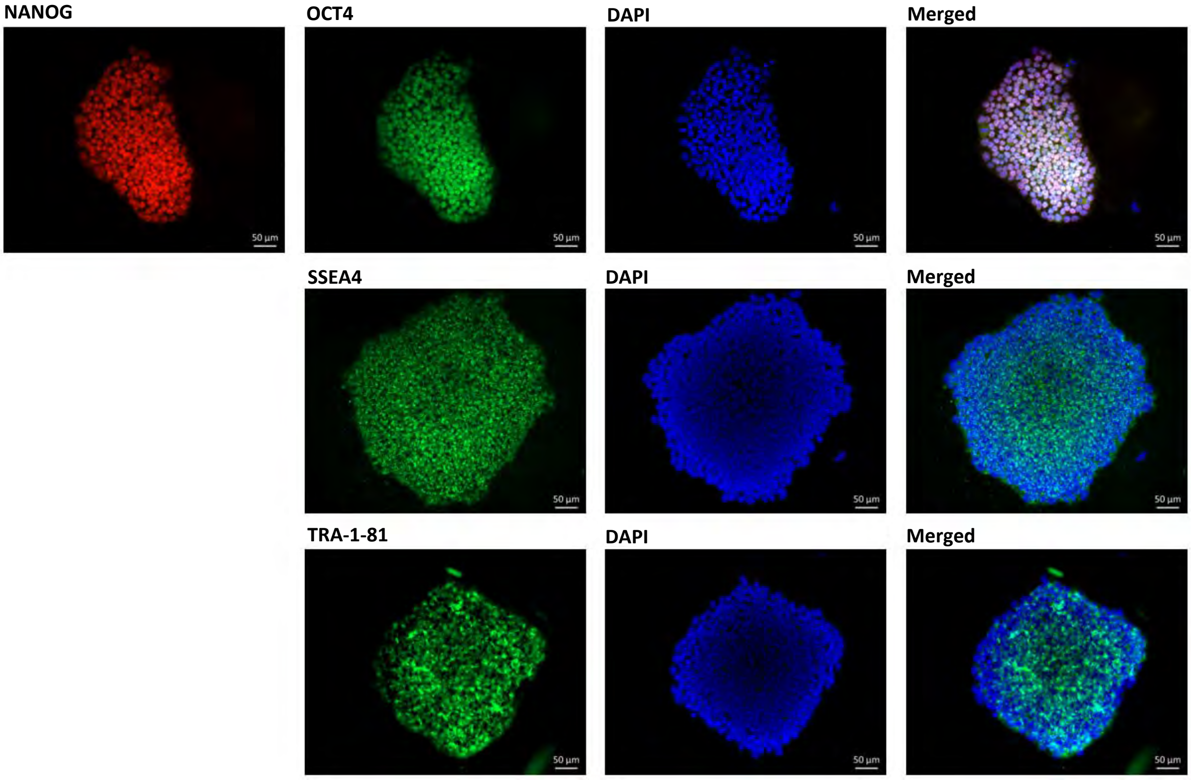

C. IPS19-00097 clone 3

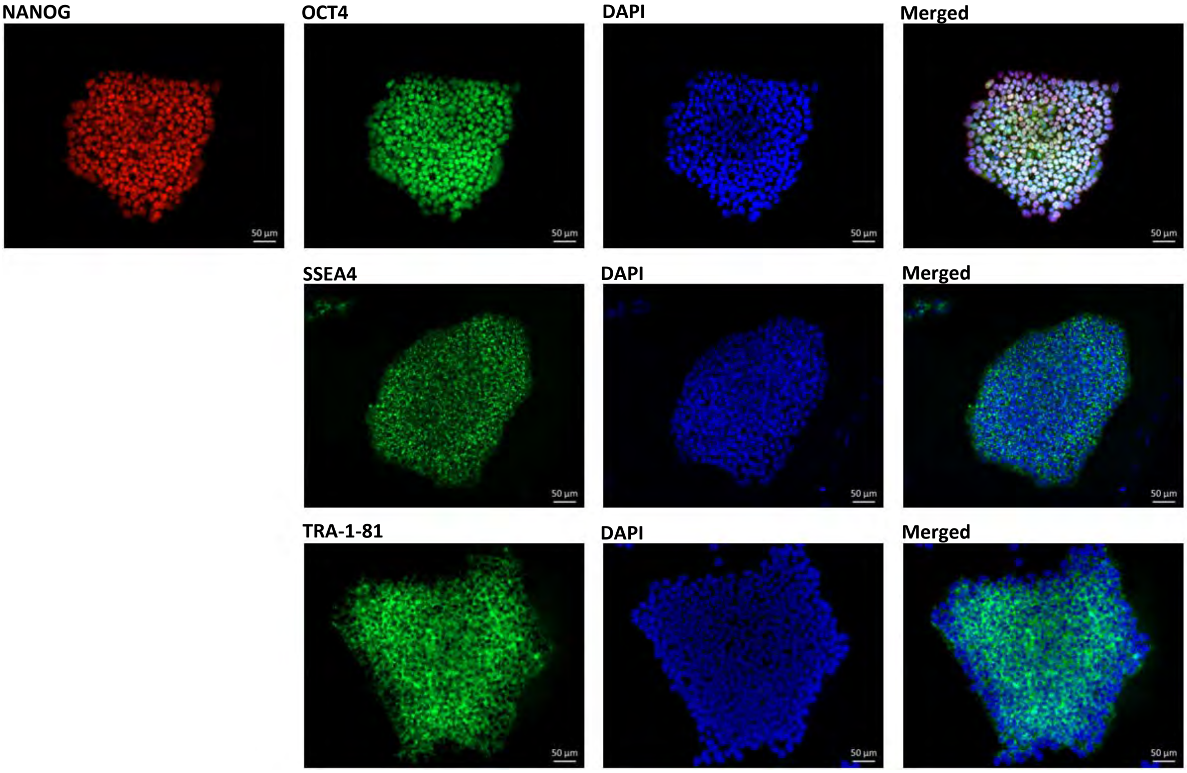

Figure 4: Immunofluorescence staining of the iPSC clones with pluripotency markers.

Three germ layer differentiation

IPS19-00097 clone 1 was differentiated into the endodermal, mesodermal and ectodermal germ layers. RNA was isolated and gene expression was checked by qPCR. Ct values are normalized with the housekeeping gene GUSB (set at 1). For each lineage two genes were assessed (Table 3). The differentiated cells were also stained for lineage-specific markers (Table 4).

Table 3: qPCR markers for three lineage differentiation

Lineage	Marker
Endoderm	FOXA2, SOX17
Mesoderm	Brachyury, HAND1
Ectoderm	PAX6, NCAM1

Table 4: ICC markers for three lineage differentiation

Lineage	Marker
Endoderm	SOX17
Mesoderm	NCAM1
Ectoderm	NESTIN

Endoderm

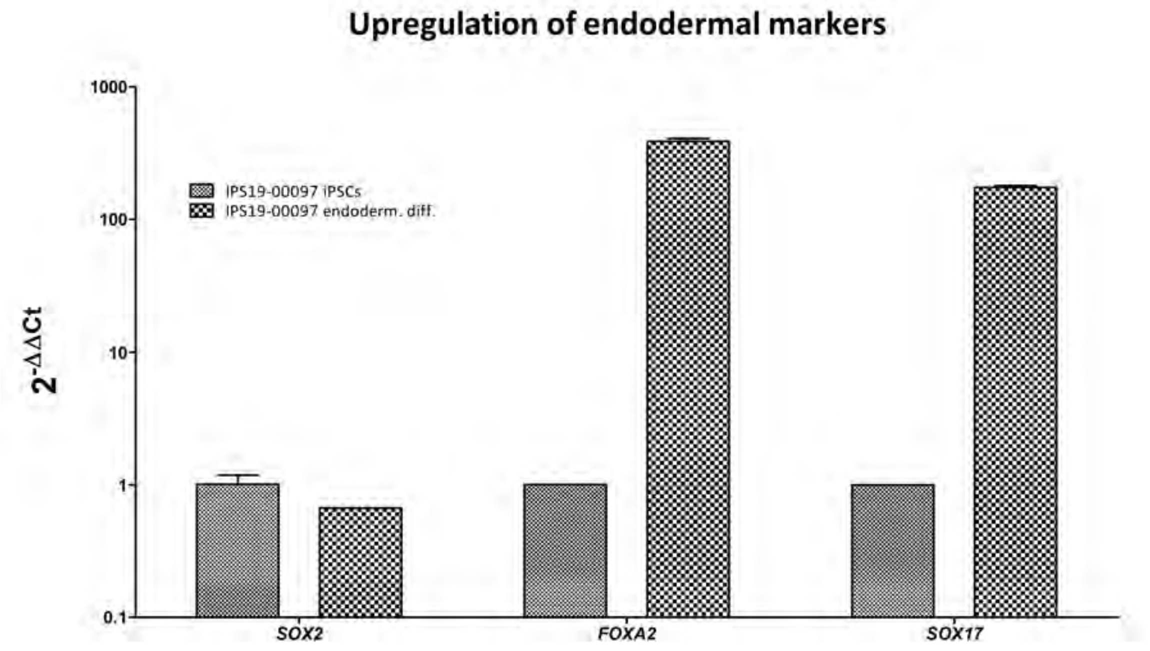

Figure 5: Expression fold difference of endoderm-specific genes in differentiated cells, compared with undifferentiated iPSCs. *SOX2* was used as a reference for pluripotency.

Mesoderm

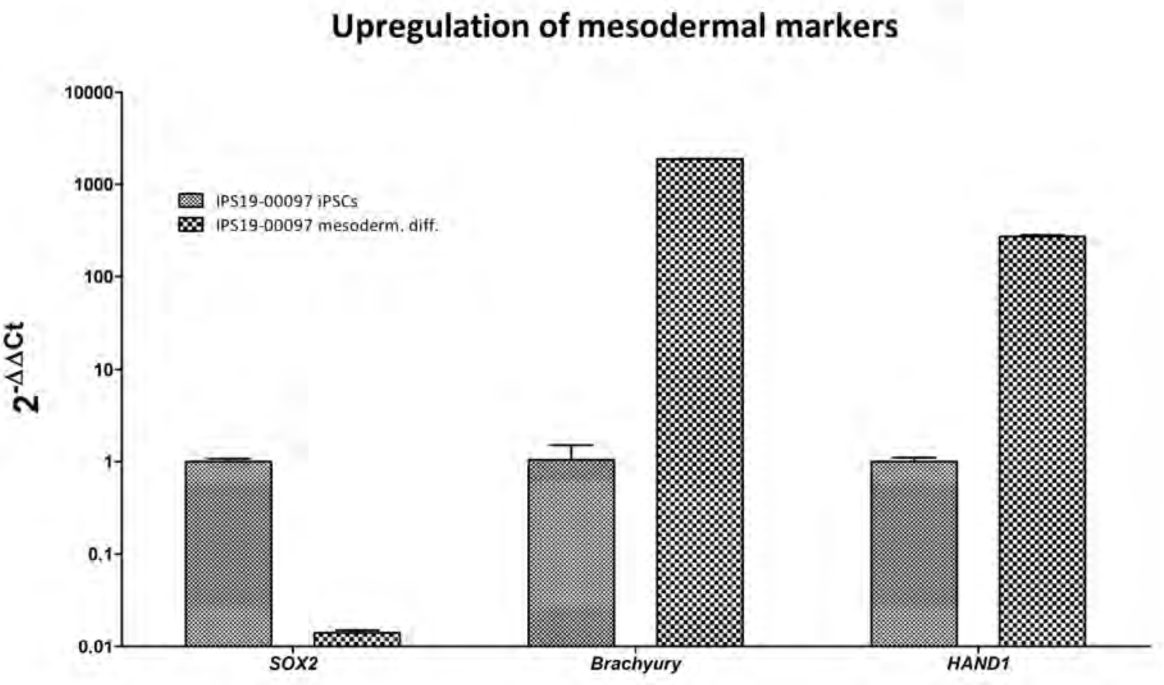

Figure 6: Expression fold difference of mesoderm-specific genes in differentiated cells, compared with undifferentiated iPSCs. *SOX2* was used as a reference for pluripotency.

Ectoderm

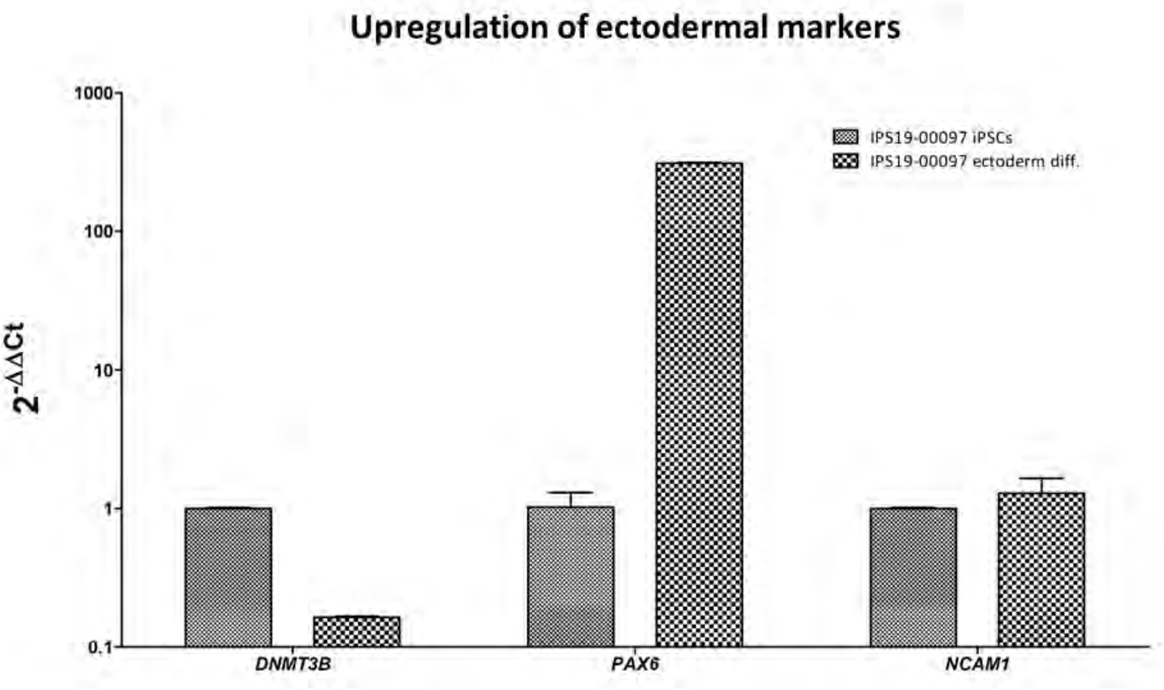

Figure 7: Expression fold difference of ectoderm-specific genes in differentiated cells, compared with undifferentiated iPSCs. *DNMT3B* was used as a reference for pluripotency.

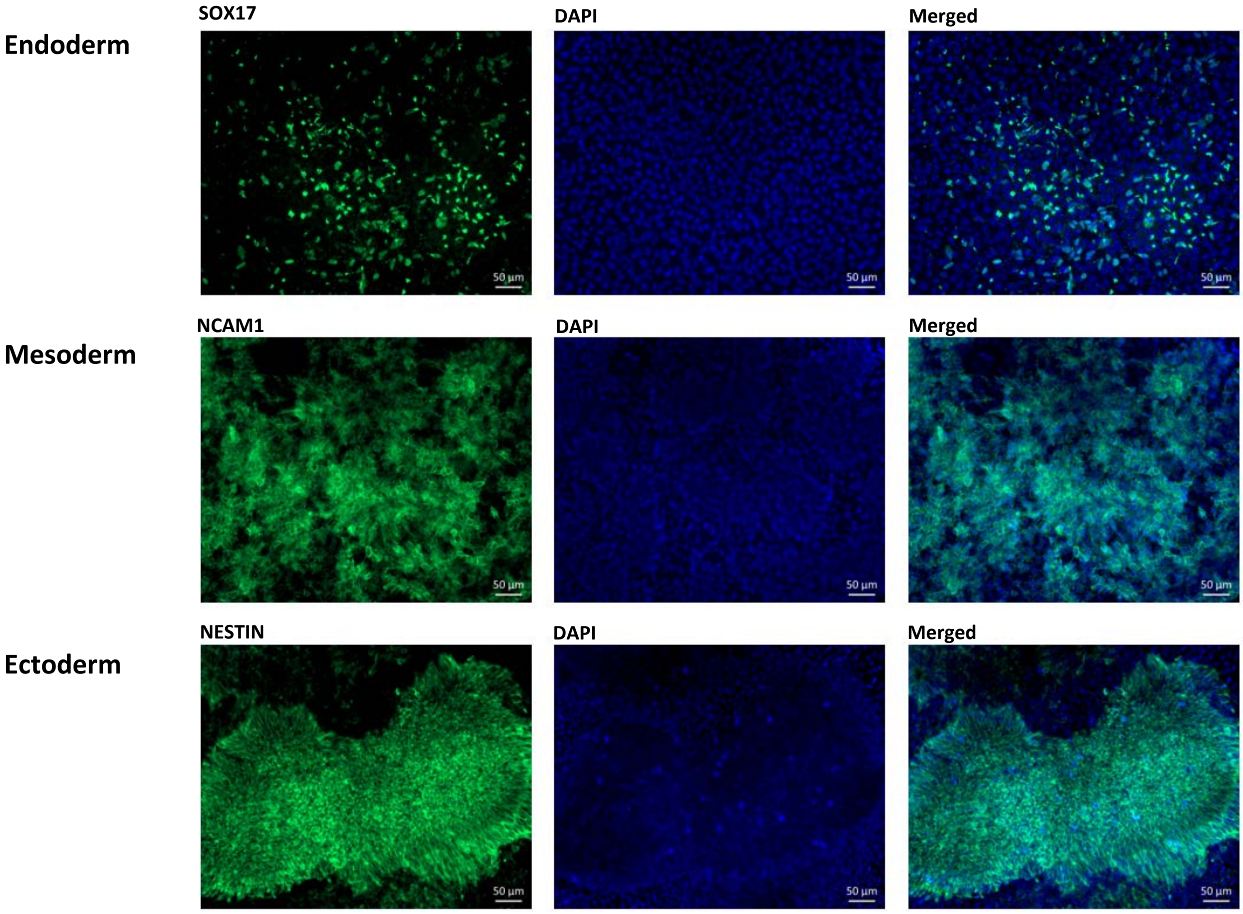

Figure 8: Immunofluorescence staining of differentiated cells showing positive signal of germlayer-specific markers.

Genetic analysis

DNA was isolated from three iPSC clones and the majority of recurrent chromosomal abnormalities reported in human embryonic stem cells and iPSCs was analysed.

Sample	Sex	Status
IPS19-00097 clone 1 P10	Female	Possibly Abnormal
IPS19-00097 clone 2 P10	Female	Normal
IPS19-00097 clone 3 P10	Female	Possibly Abnormal

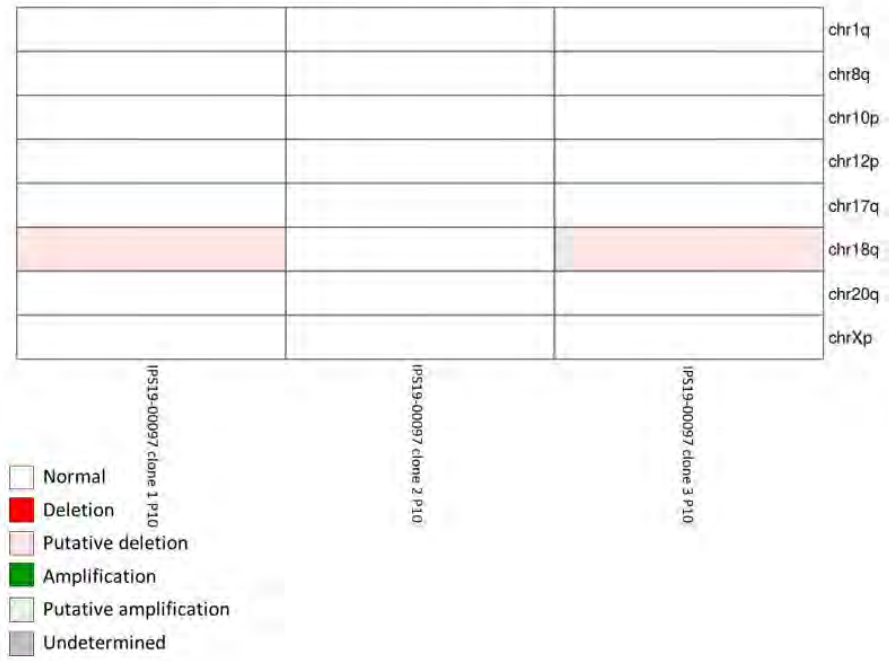

Figure 9: Summary of the genetic analysis

For further experiments it is suggested to use IPS19-00097 clone 2. It is suggested to check IPS19-00097 clone 1 and 3 at a later passage to assess whether there is indeed a mutant clone in the culture that expands over time.

More detailed results are on request.

Stem Cell Technology Center Genetica

Certificate of Analysis 2019

Invoice number: SCTC2019-00020

Name investigator: Anneke den Hollander
Cell line number: IPS19-00013
Project name: VICI

Table 1: Information on the reprogrammed cell line

**Information cell line:**	
Product description	PBMCs nucleofected with episomal vectors containing the genes OCT3/4, SOX2, KLF4, L-MYC, LIN28
Parental cell line	HEP19-00031
Parental cell type	PBMCs
Diagnosis	AMD-CON
Mutation	N/A*
Number of clones	3
Passage (P) of iPSCs reported at submission	P10
Culture medium	Essential 8 Flex medium
Culture coating	Matrigel
Feeders during reprogramming	Mouse Embryonic Fibroblasts (MEFs)
Passage method	0.5 mM EDTA
Protocols in Q-portal	046588; 046591

Table 2: Information on the characterization of the reprogrammed cell line

**Test description:**	**Test method:**	**Test specification:**	**Result:**
Activation of stem cell markers	qPCR	Upregulation of *SOX2, LIN28, NANOG, DNMT3B* compared with PBMCs	Pass
Expression of stem cell markers	Immunocytochemistry	Expression of OCT4, NANOG, SSEA4, TRA-1-81	Pass
Mycoplasma test	PCR	Negative	Pass
Three lineage differentiation	Differentiation assay	Upregulation of germlayer-specific genes	Pass
hPSC genetic analysis	qPCR	Detection of recurrent chromosomal abnormalities	See results in last page

* N/A: Not Applicable

A B C

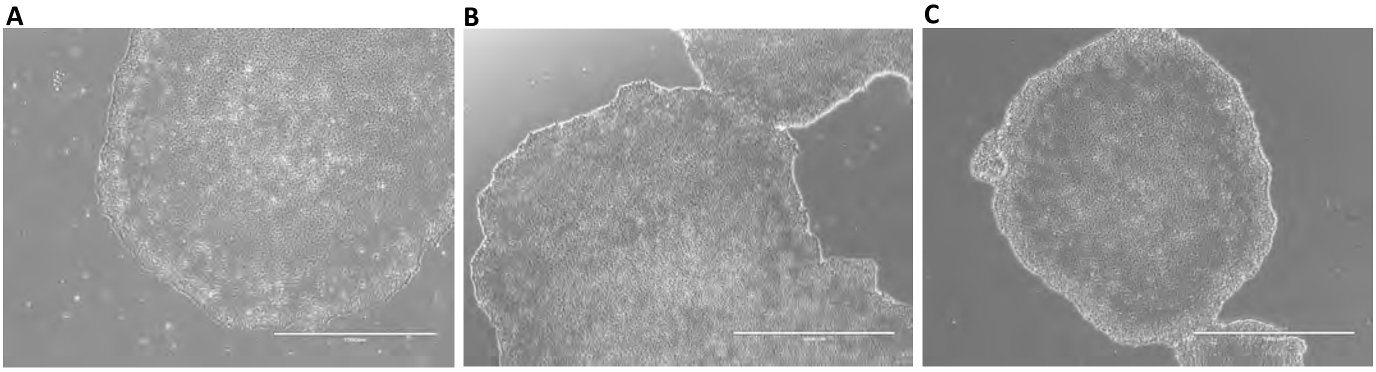

Figure 1: Cells prior to freezing. A - C, clone 1, clone 2 and clone 3 at passage 10, respectively. Scale bar = 1000 µm.

Activation of stem cell markers

All clones were assessed for activation of stem cell markers before freezing. RNA was isolated and gene expression was assessed by quantitative reverse transcription PCR. Ct values were normalized with the housekeeping gene GUSB (set at 1).

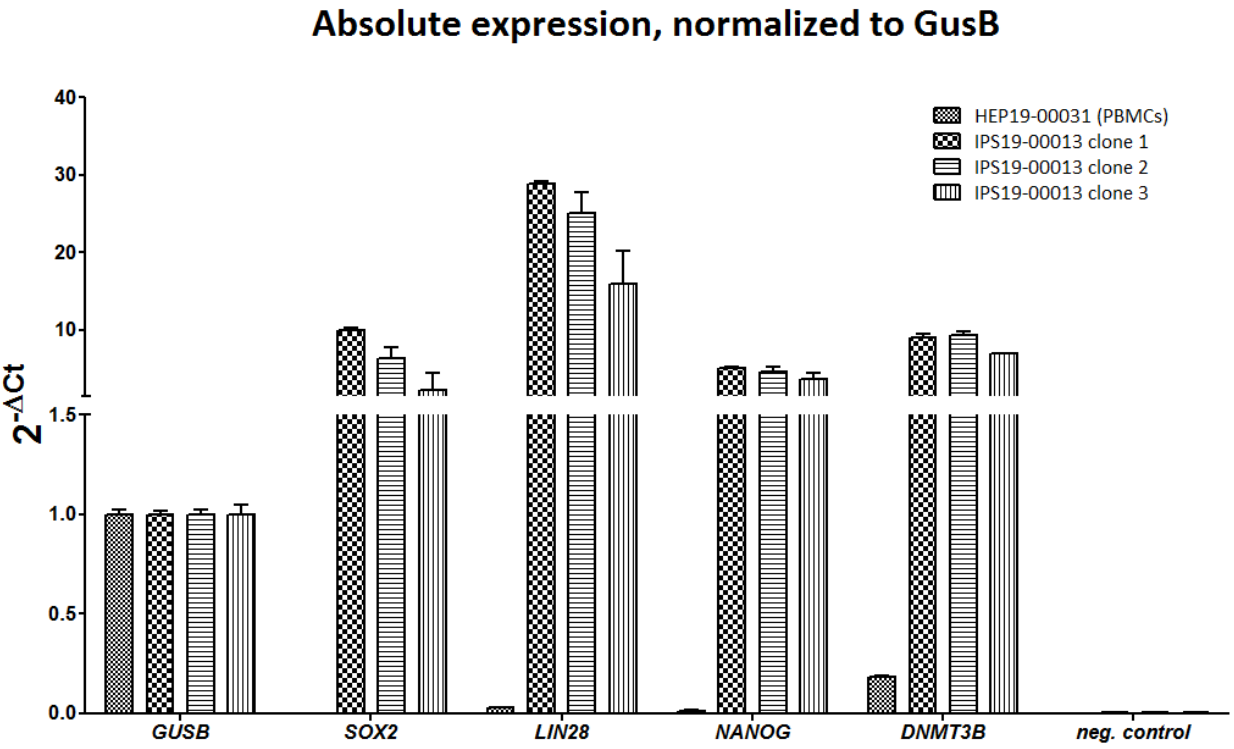

Figure 2: Gene expression of three iPSC clones compared with the parental PBMCs (ΔCt).

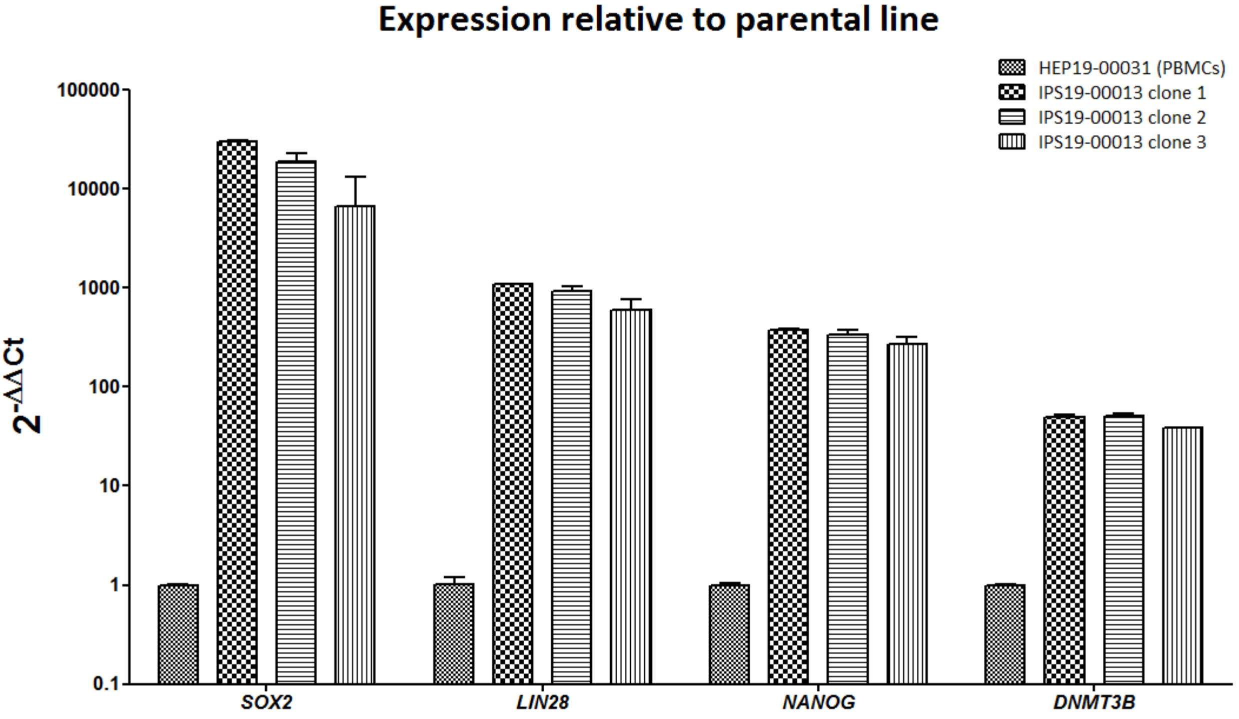

Figure 3: Pluripotency gene upregulation after reprogramming (ΔΔCt). The expression fold difference of the iPSC clones is relative to the parental PBMCs.

Expression of stem cell markers

Undifferentiated iPSC clones were stained for the nuclear markers NANOG and OCT4 and surface antigens SSEA4 and TRA-1-81. All markers are expressed in human pluripotent stem cells.

A. IPS19-00013 clone 1 P10

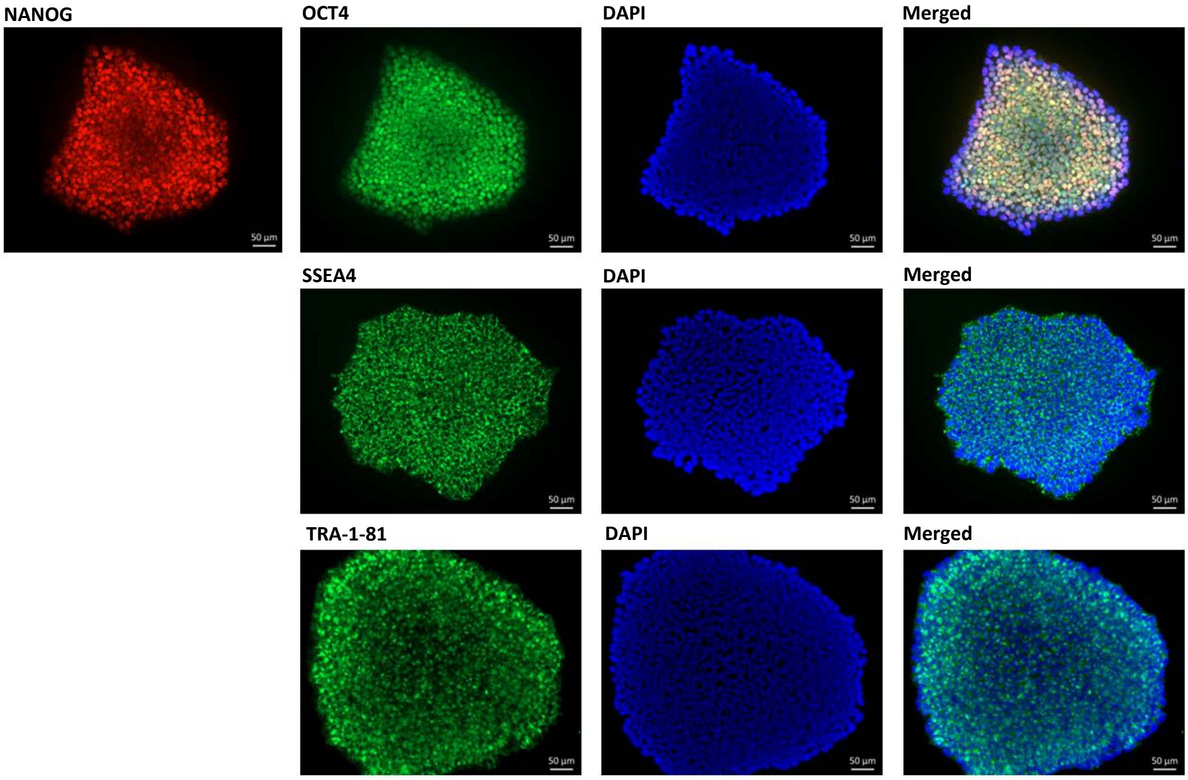

B. IPS19-00013 clone 2 P10

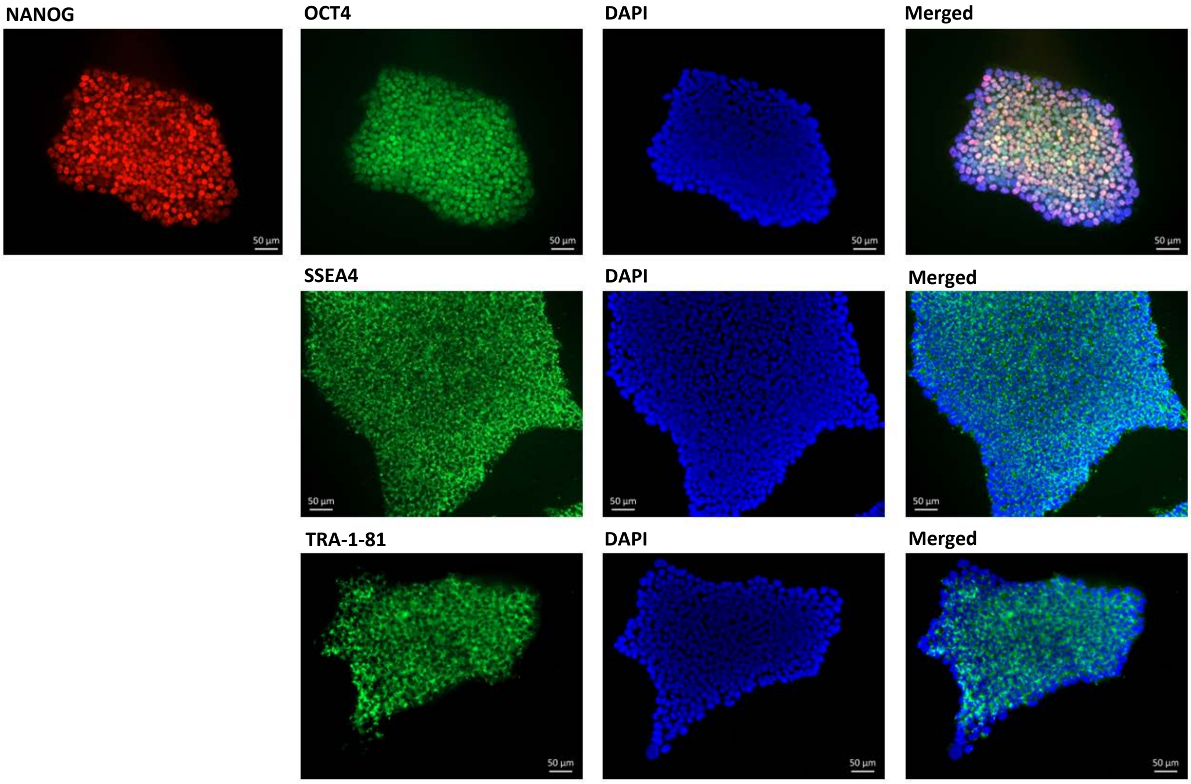

C. IPS19-00013 clone 3 P10

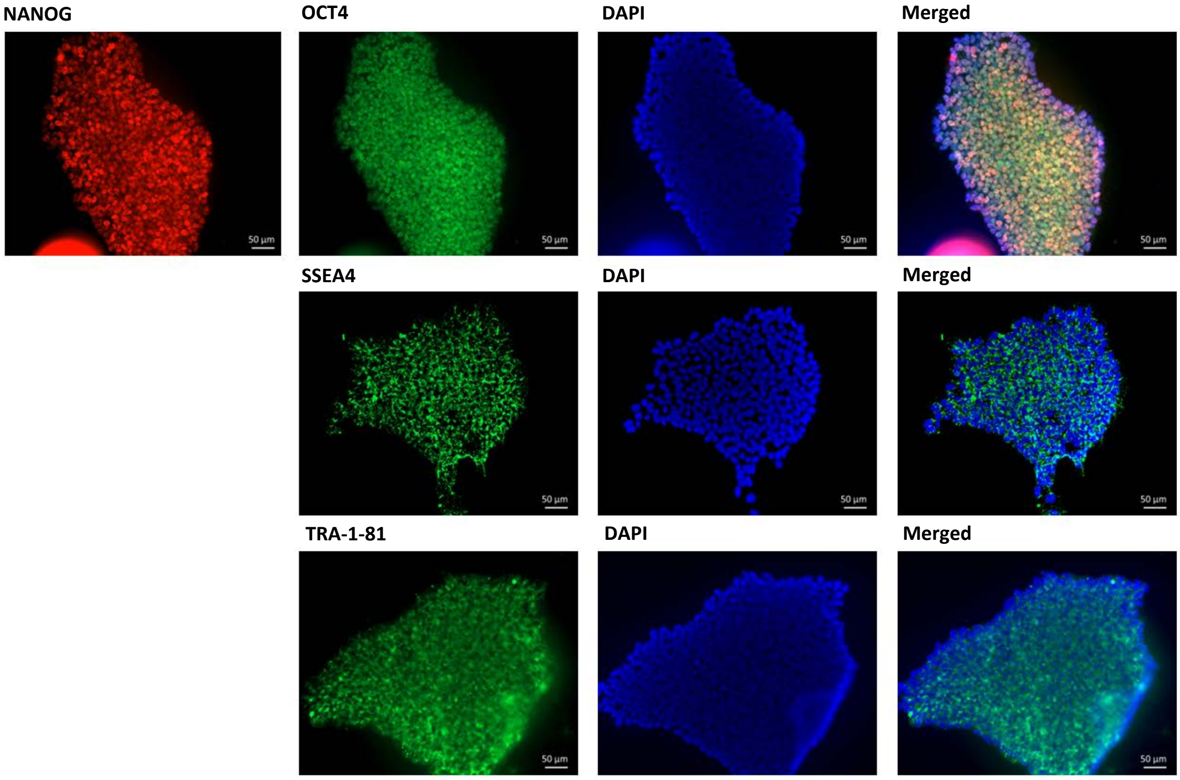

Figure 4: Immunofluorescence staining of the iPSC clones with pluripotency markers.

Three germ layer differentiation

iPSC clones were differentiated into the endodermal, mesodermal and ectodermal germ layers. RNA was isolated and gene expression was checked by qPCR. Ct values are normalized with the housekeeping gene GUSB (set at 1). For each lineage two genes were assessed (Table 3). The differentiated cells were also stained for lineage-specific markers (Table 4).

Table 3: qPCR markers for three lineage differentiation

Lineage	Marker
Endoderm	FOXA2, SOX17
Mesoderm	Brachyury, HAND1
Ectoderm	PAX6, NCAM1

Table 4: ICC markers for three lineage differentiation

Lineage	Marker
Endoderm	SOX17
Mesoderm	NCAM1
Ectoderm	NESTIN

Endoderm

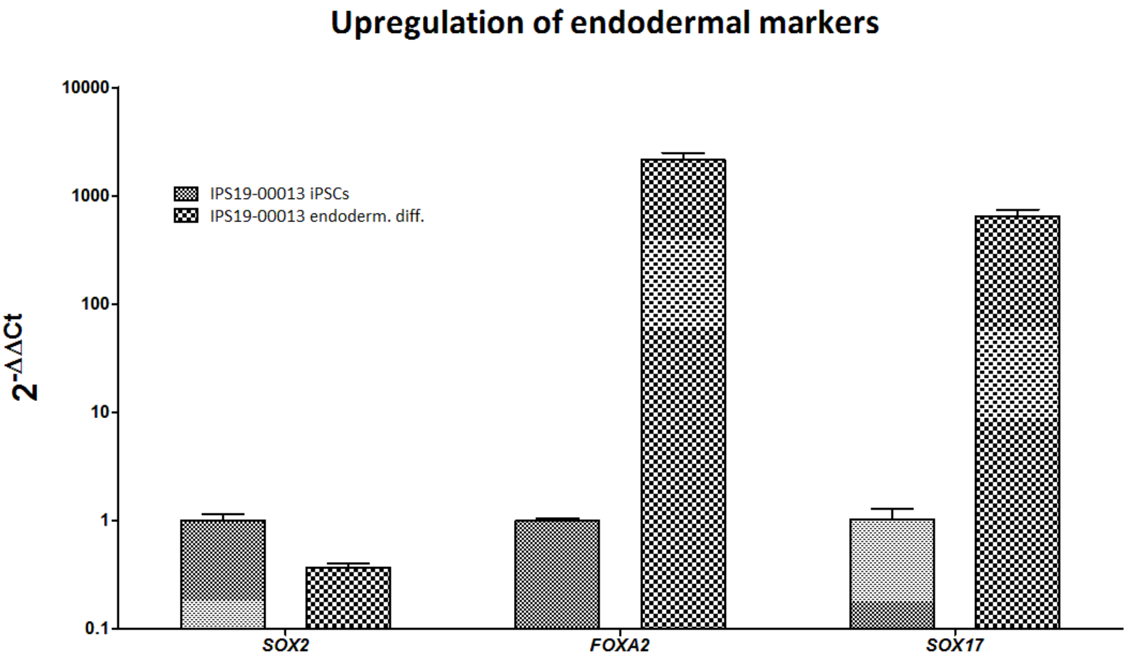

Figure 5: Expression fold difference of endoderm-specific genes in differentiated cells, compared with undifferentiated iPSCs. *SOX2* was used as a reference for pluripotency.

Mesoderm

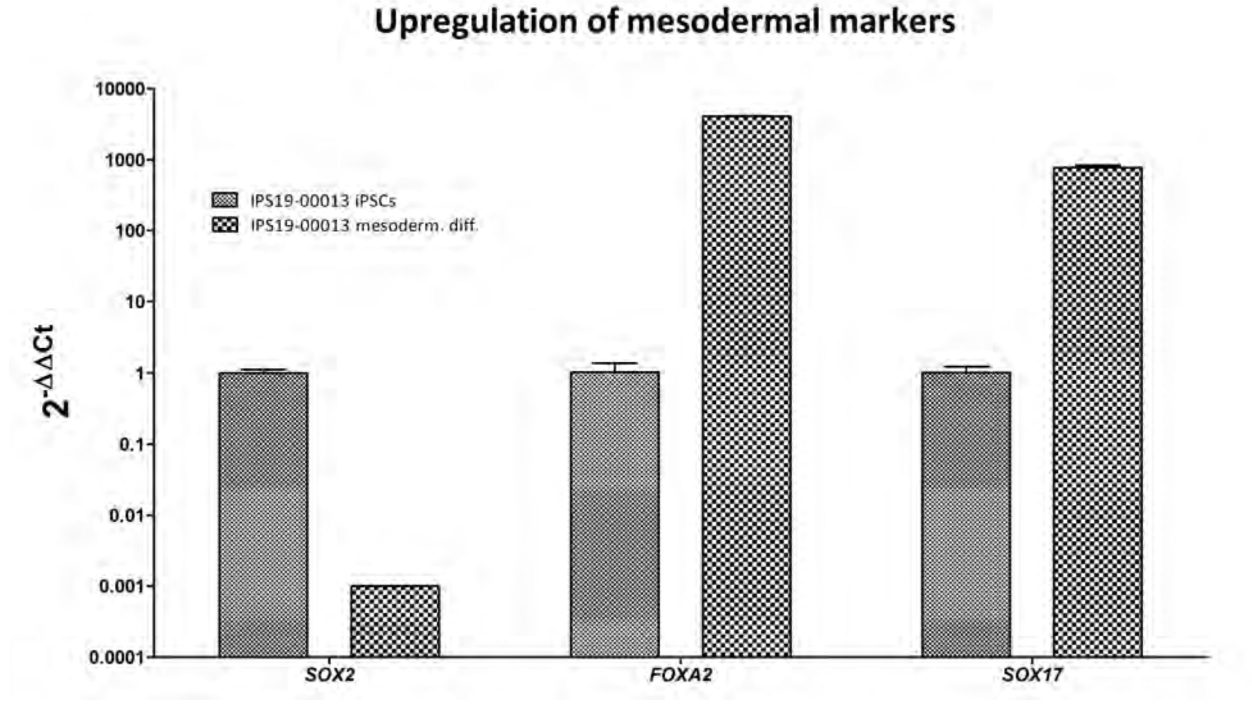

Figure 6: Expression fold difference of mesoderm-specific genes in differentiated cells, compared with undifferentiated iPSCs. *SOX2* was used as a reference for pluripotency.

Ectoderm

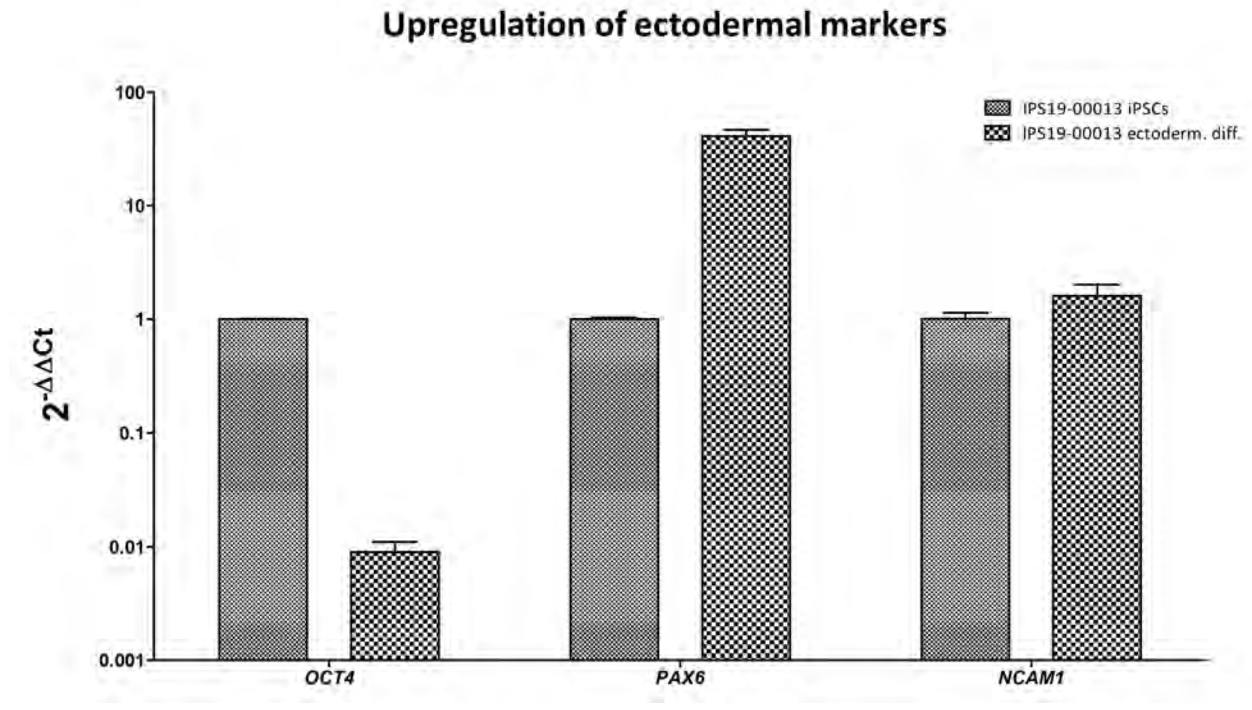

Figure 7: Expression fold difference of ectoderm-specific genes in differentiated cells, compared with undifferentiated iPSCs. *OCT4* was used as a reference for pluripotency.

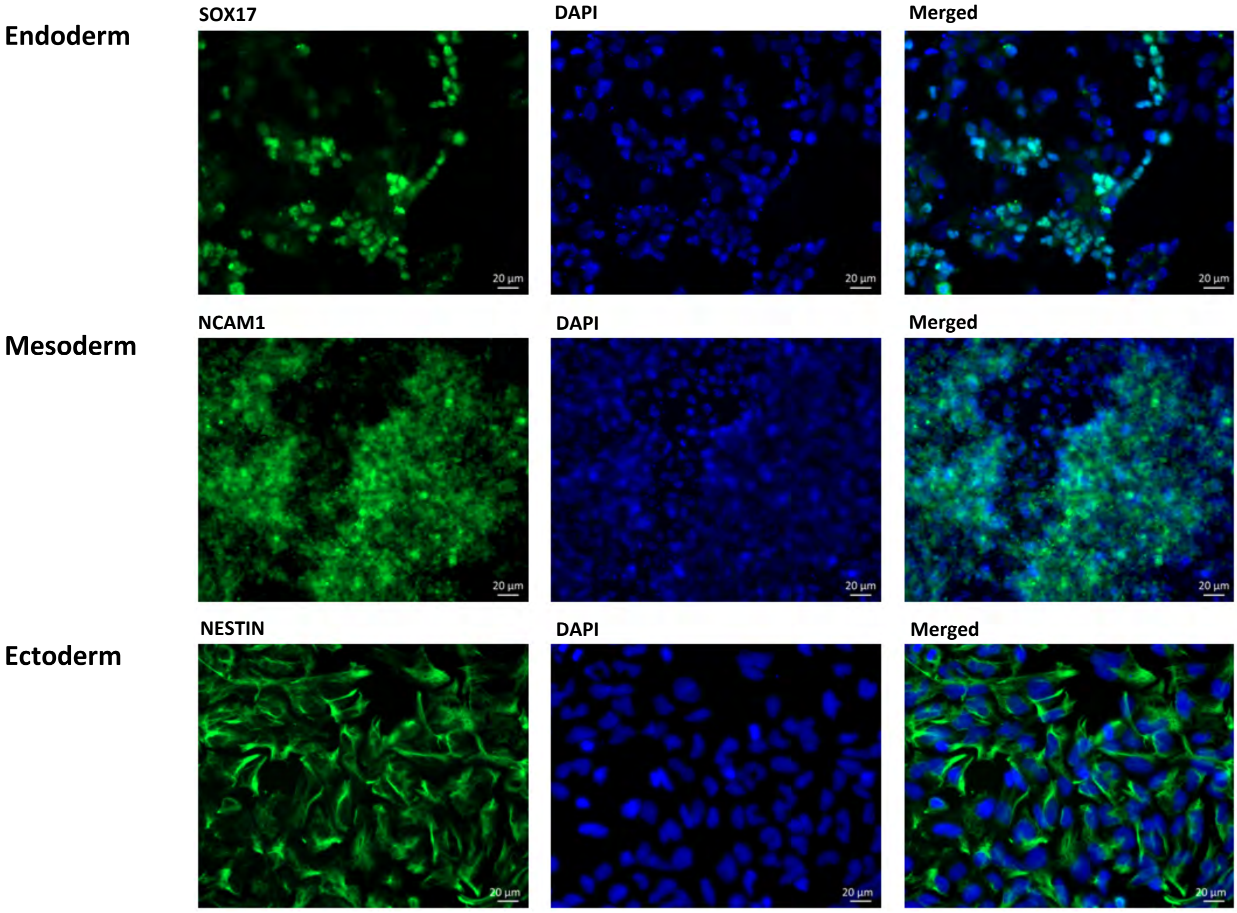

Figure 8: Immunofluorescence staining of differentiated cells showing positive signal of germ layer-specific markers.

Genetic analysis

DNA was isolated from three iPSC clones before analyzing the majority of recurrent chromosomal abnormalities reported in human embryonic stem cells and induced pluripotent stem cells.

Sample	Sex	Status
IPS19-00013 clone 1	Female	Possibly Abnormal
IPS19-00013 clone 2	Female	Normal
IPS19-00013 clone 3	Female	Normal

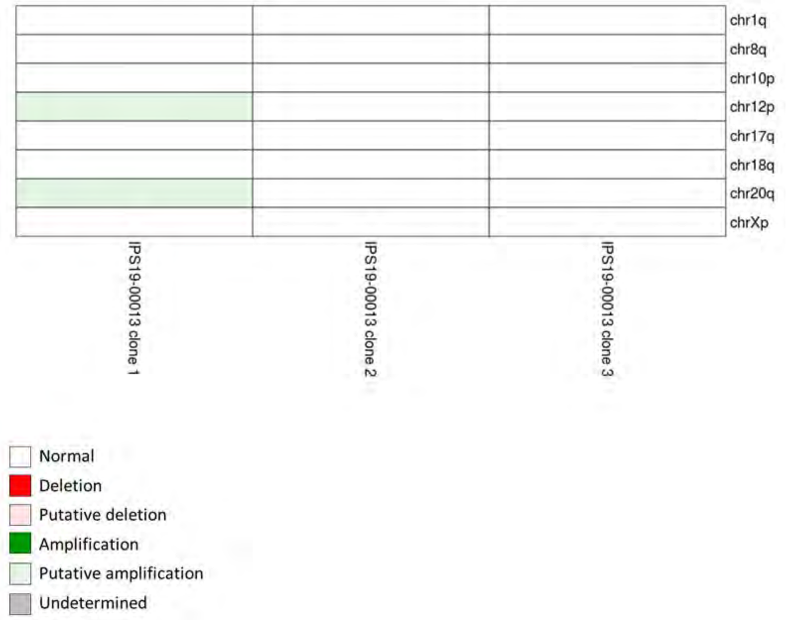

Figure 9: Summary of the genetic analysis

Conclusion:
It is suggested to check IPS19-00013 clone 1 at a later passage to assess whether there is indeed a mutant clone in the culture that expands over time. More detailed results are on request.
